# Effects of Lifestyle Modification on Intrapancreatic Fat Deposition: A Systematic Review and Meta-Analysis

**DOI:** 10.1007/s13679-026-00737-0

**Published:** 2026-07-20

**Authors:** Saeed Ahmad, Hyun Hee Sul, Pojsakorn Danpanichkul, Matheus Souza, Maxim S. Petrov, The Global Intrapancreatic FaT (GIFT) Alliance

**Affiliations:** 1 Beth Israel Deaconess Medical Center, Harvard Medical School, Boston, MA USA; 2 Department of Internal Medicine, Jersey City Medical Center/Rutgers Health, Jersey City, New Jersey USA; 3 Department of Internal Medicine, Texas Tech University Health Sciences Center, Lubbock, TX USA; 4 Department of Internal Medicine, Federal University of Rio de Janeiro, Rio de Janeiro, RJ Brazil; 5 School of Medicine, University of Auckland, Auckland, New Zealand

**Keywords:** Intrapancreatic fat deposition, Lifestyle modification, Diet, Exercise

## Abstract

**Background & Aims:**

Lifestyle modification is a well-established approach for improving metabolic health and reducing ectopic fat accumulation in the liver. The Melbourne Consensus has recently highlighted intrapancreatic fat deposition (IPFD) as an important fat depot, playing a key role in the development of both endocrine and exocrine pancreatic diseases. Nevertheless, the existing literature on the impact of lifestyle modification on IPFD has not been systematically synthesized. We conducted a comprehensive systematic review and meta-analysis to assess the effects of lifestyle modification on IPFD.

**Methods:**

A systematic literature search was conducted in PubMed and Embase databases to identify interventional studies evaluating the effects of lifestyle modification on IPFD measured by magnetic resonance-based techniques. Data were meta-analyzed using a random-effects model. Statistical heterogeneity was assessed using the I² statistic, and publication bias was evaluated with Egger’s test.

**Results:**

A total of 23 studies were included. Lifestyle modification resulted in a significant reduction in IPFD (standardized mean difference [SMD] -0.26, 95% CI -0.36 to -0.16; p<0.0001), with low heterogeneity (I^2^=12.9%). The pooled absolute reduction in MRI-measured IPFD was −1.02 % (95% CI −1.30 to −0.73; p<0.0001), with no evidence of heterogeneity (I^2^=0%). Subgroup analyses showed the largest numerical reduction in IPFD with combined diet-and-exercise interventions (SMD -0.41, 95% CI -0.81 to -0.02, I^2^=29.4%), followed by exercise alone (SMD -0.26, 95% CI -0.36 to -0.15, I^2^=0%) and diet alone (SMD -0.22, 95% CI -0.37 to -0.08, I^2^=27.9%). However, differences between intervention types were not statistically significant (p=0.40). There was no evidence of publication bias (p = 0.78).

**Conclusion:**

Lifestyle modification is an effective non-pharmacological approach for reducing IPFD, with combined diet-and-exercise interventions showing the largest numerical effect. These findings support the potential of structured lifestyle modification programs to reduce IPFD and may have implications for the prevention and management of pancreatic diseases.

**Graphical Abstract:**

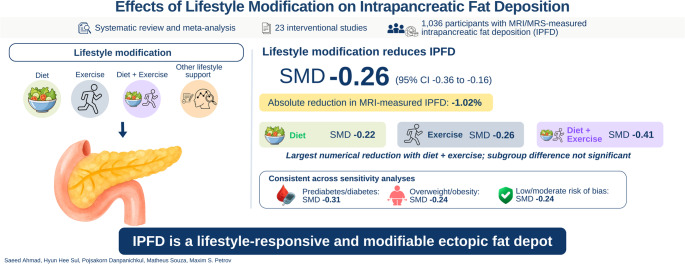

**Supplementary Information:**

The online version contains supplementary material available at 10.1007/s13679-026-00737-0.

## Introduction

Intrapancreatic fat deposition (IPFD) is increasingly recognized as a clinically relevant ectopic fat depot, and the Melbourne Consensus has substantially advanced the field by establishing the first framework for its conceptualization.[1, 2] Once considered a passive consequence of obesity, IPFD is now viewed as an active contributor to pancreatic and metabolic disease pathways.[1, 3–6] Advances in abdominal imaging, particularly chemical shift-encoded magnetic resonance imaging (MRI), have enabled precise quantification of IPFD, prompting a paradigm shift in the field.[7, 8] The PANDORA (PANcreatic Diseases Originating from intRa-pancreatic fAt) hypothesis posits that excessive IPFD plays a causative role in the pathogenesis of endocrine and exocrine pancreatic diseases, including type 2 diabetes mellitus (T2DM), pancreatitis, and pancreatic cancer.[3] This hypothesis has been strongly supported by evidence from recent longitudinal studies.[9–16] Excessive IPFD affects approximately one in five people in the general population[9, 17, 18] and is even more common among high-risk groups, including individuals with T2DM, obesity, metabolic dysfunction-associated steatotic liver disease (MASLD), and cardiovascular disease.[6, 9, 19, 20].

Management of cardiometabolic disease heavily relies on lifestyle modification as a cornerstone, particularly in the early phases.[21–24] However, whether the benefits of lifestyle modification extend to the pancreas is less well characterized. Interventional studies evaluating hypocaloric diets, structured exercise programs, or combined lifestyle strategies have produced conflicting results, reporting changes in IPFD ranging from marked reductions to no effect.[25–30] These studies are often limited by small sample sizes, heterogeneous populations, and variable interventions and follow-up durations. Moreover, it remains unclear whether changes in IPFD parallel reductions in body mass index (BMI) or hepatic fat—distinctions that may help differentiate shared versus organ-specific mechanisms of ectopic fat remodeling.

The primary aim of this study was to systematically evaluate the overall effect of lifestyle modification on IPFD. The secondary aim was to quantify and compare IPFD changes following different types of lifestyle interventions. The tertiary aim was to assess the influence of relevant covariates on the observed effects.

## Methods

### Study Design and Search Strategy

The present systematic review was designed in accordance with the Preferred Reporting Items for Systematic Reviews and Meta-Analyses (PRISMA) guidelines and the Meta-analysis Of Observational Studies in Epidemiology (MOOSE) guidelines.[31, 32] The study protocol was prospectively registered in the International Prospective Register of Systematic Reviews (PROSPERO) (CRD420251232097). A systematic literature search of the PubMed and Embase databases was conducted from inception through February 25, 2026, to identify studies evaluating the effects of lifestyle modifications on IPFD. Lifestyle modification was defined as non-pharmacological, non-surgical approaches intended to induce beneficial changes in body composition or metabolic outcomes, regardless of whether energy restriction was involved. Behavioral counseling and education were included when explicitly aimed at modifying lifestyle behaviors. The full electronic search strategy is provided in the Supplementary Methods. To ensure comprehensive study identification, we additionally screened the reference lists of all eligible articles and relevant reviews and consulted the authors’ personal literature collections.

### Eligibility Criteria

Two investigators (S.A. and H.H.S.) independently screened titles and abstracts to identify potentially relevant studies according to predefined eligibility criteria. Full texts of selected studies were then reviewed for final inclusion. Discrepancies were resolved through discussion with a third investigator (M.S.). Included studies were required to meet all three of the following principal inclusion criteria: (1) interventional studies conducted in humans, including specific study arms of randomized controlled trials (RCTs) and non-randomized interventional studies; (2) studies examining the effects of any lifestyle intervention on changes in IPFD from baseline to post-intervention, measured by magnetic resonance-based techniques (e.g., MRI and magnetic resonance spectroscopy [MRS]); (3) studies providing sufficient information to enable quantitative synthesis of IPFD data (i.e., baseline and post-intervention values or baseline values with reported change in IPFD), irrespective of whether IPFD was a primary, secondary, or exploratory outcome. For RCT study arms, only arms (intervention or control) involving a lifestyle modification were eligible for inclusion. When multiple eligible arms existed within a single RCT, each was considered separately. Post hoc analyses combining study arms in RCTs were considered eligible if the original arms had been previously shown to have no significant differences. Post-intervention IPFD measurements had to be obtained at the time of completion of the lifestyle intervention; subsequent maintenance period was not considered part of the intervention. Exclusion criteria comprised non-interventional studies (e.g., studies relying on self-reported dietary intake), animal studies, abstracts, and non-original publications (e.g., guidelines, commentaries, editorials, and reviews). Studies evaluating overfeeding interventions were not classified as lifestyle interventions and were therefore excluded from the systematic review.[33, 34] When multiple publications derived from the same cohort were identified, only one study was included in the meta-analysis to avoid duplicate data.

### Data Extraction and Risk of Bias Assessment

The following items of interest were extracted from each study: (1) study characteristics: first author, year, country, study design, eligibility criteria, IPFD assessment method, description of intervention; (2) population characteristics at baseline: sample size, age, sex, baseline IPFD, body weight (BW), BMI; and (3) study results: loss to follow-up, intention-to-treat or per-protocol analysis, and changes in IPFD, BW, BMI, and liver fat content (LFC). All relevant data were recorded in predefined tables. Data reported as graphs or bar charts were also manually extracted using WebPlotDigitizer (version 4.7). Two investigators (H.H.S. and S.A.) independently evaluated the risk of bias in eligible studies using the Cochrane Risk of Bias 2 tool (RoB 2) for RCTs and Risk of Bias in Non-randomized Studies of Interventions (ROBINS-I) tool for non-randomized studies.[35, 36] Discrepancies were resolved through discussion with a third investigator (M.S.).

### Statistical Analysis

All analyses were conducted in RStudio (version 4.4.0), and a two-sided p-value < 0.05 was considered statistically significant. The primary outcome was the standardized mean difference (SMD) in IPFD following lifestyle modification, enabling harmonization across magnetic resonance-based modalities and protocols. Mean differences (MDs) were additionally estimated in MRI-based studies to quantify changes in IPFD on a clinically interpretable percentage-point scale, with analyses further stratified by lifestyle intervention type. Pooled SMDs and MDs were calculated using random-effects models with restricted maximum likelihood estimation of between-study variance (τ^2^) and Hartung-Knapp-adjusted confidence intervals. When necessary, means and standard deviations (SDs) in primary studies were estimated from medians and interquartile ranges or ranges using the transformations described elsewhere.[37, 38] When post-intervention IPFD values were not directly reported, post-intervention means were reconstructed by adding the reported mean change to the corresponding baseline mean. Missing post-intervention SDs were imputed using the method described by Furukawa et al.[39] by borrowing pooled post-intervention SDs from studies with available SD data that used comparable IPFD assessment methods and interventions. To avoid introducing uncertainty into moderator analyses, data requiring imputation of post-intervention SDs were excluded from meta-regression analyses.

Heterogeneity was assessed using the I^2^ statistic (low: 0–50%; moderate: 50–75%; and high: 75–100%) and τ^2^. Publication bias was evaluated through visual inspection of funnel plots and formally tested using Egger’s regression test. Subgroup analyses were conducted based on the type of lifestyle intervention, method of IPFD measurement, and follow-up duration. Within the dietary intervention subgroup, we further performed stratified analyses according to dietary strategy (energy-restricted interventions vs. isocaloric/weight-maintenance dietary interventions). Sensitivity analyses were conducted by restricting the dataset to studies in patients with prediabetes or T2DM, studies in populations with overweight or obesity, and studies assessed as having a low risk of bias. Robustness was further assessed using a leave-one-out analysis. Univariate meta-regressions were conducted to examine the influence of study-level variables—percentage of female, baseline BW, baseline IPFD, % weight loss, SMDs in BMI and LFC—on changes in IPFD following lifestyle interventions. Multivariable meta-regression models were also constructed based on study-level variables that were significant in univariate analyses.

## Results

### Study Characteristics

**Fig. 1:**
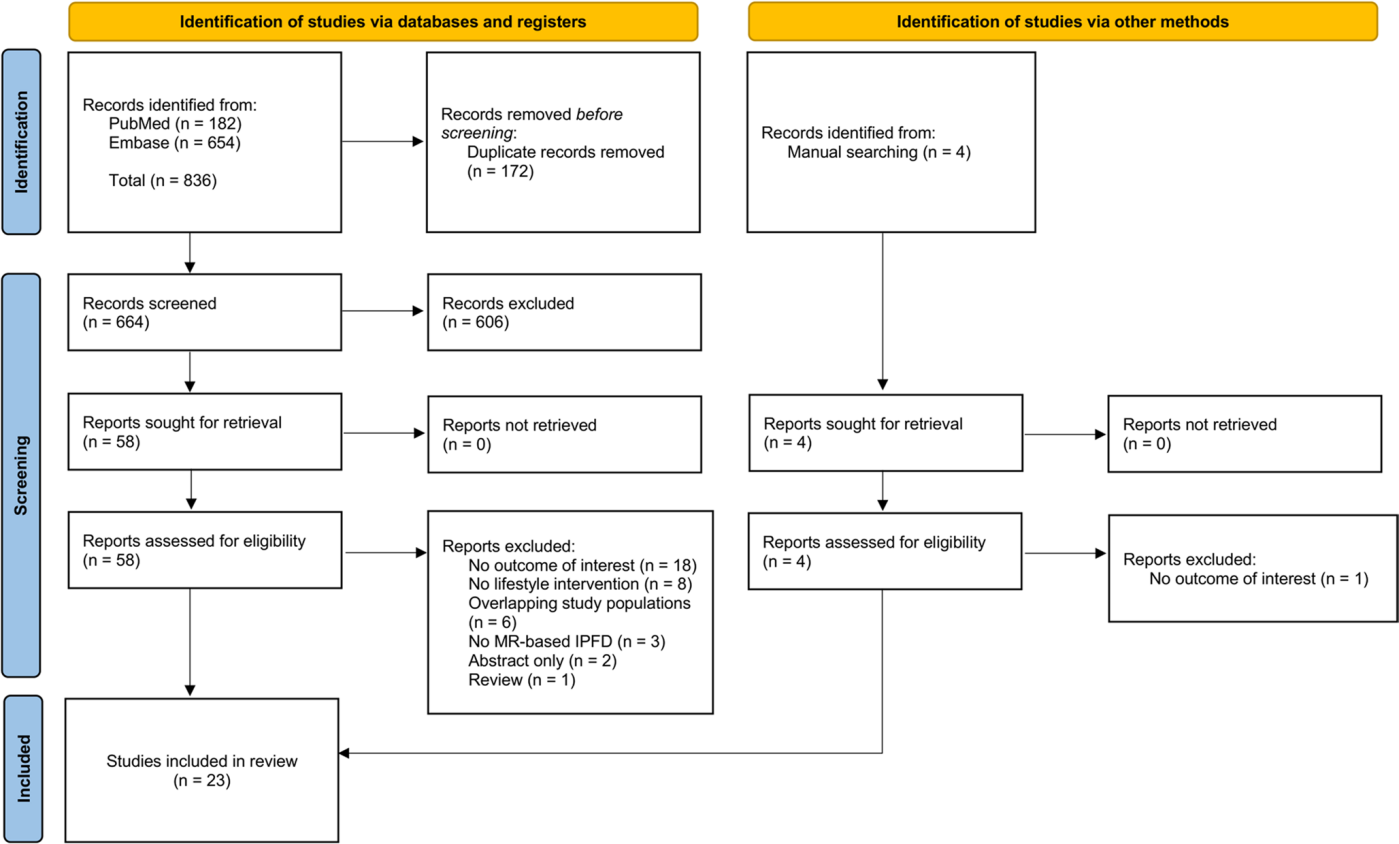
PRISMA flow diagram. PRISMA, preferred reporting items for systematic reviews and meta-analyses

The initial search yielded 836 records. After the removal of 172 duplicates, 664 records were screened based on titles and abstracts. Full-text assessment was conducted for 58 studies, of which 20 met all prespecified eligibility criteria.[25–27, 29, 40–55] We also identified 3 studies via other methods.[56–58] Finally, 23 studies were included in this systematic review (Fig. 1).[25–27, 29, 40–58] The risk of bias assessment of all included studies is summarized in Table S1. Of the 23 included studies, 13 evaluated diet-only interventions[26, 29, 40–49, 57], 5 examined exercise-only interventions[25, 50–52, 56], 4 assessed combined diet and exercise programs[27, 53, 54, 58], and one evaluated other combined lifestyle modification[55]. Intervention durations ranged from 2 to 26 weeks (Table 1). IPFD was predominantly assessed using MRI (*n* = 20) [25–27, 29, 40–48, 52–58] with 3 studies employing MRS[49–51]. Additional study characteristics are detailed in Table S2.

**Table 1:** Summary of studies examining the effects of lifestyle modification on IPFD

First author (year)	Location	Design	Population	Intervention(s)	Duration	Analysis type	IPFD measurement
Diet-only interventions							
Lim et al.[40] (2011)	UK	Prospective interventional study	11 adults with T2DM	VLCD ~ 600 kcal/d (Optifast + vegetables)	8 weeks	PP	MRI
Rossi et al.[41] (2012)	Italy	Prospective interventional study	24 adults with obesity	VLCD − 500 kcal/d (62% CHO)	13–26 weeks	PP	MRI
Steven et al.[42] (2016)	UK	Prospective interventional study	30 adults with T2DM (29 completed)	VLCD 624–700 kcal/d (Optifast + vegetables)	8 weeks	PP	MRI
Taylor et al.[43] (2018)	UK	RCT sub study (responders vs. non-responders)	58 adults with T2DM	Formula diet 825–853 kcal/d (12–20 w)	12–20 weeks	PP	MRI
Jiang et al.[29] (2019)	Germany	Post hoc analysis of both arms from an RCT	137 non-diabetic overweight/obese adults	Calorie restriction (intermittent/continuous/control pooled)	12 weeks	PP	MRI
Skytte et al.[44] (2019)	Denmark	Crossover RCT	28 adults with T2DM (27 completed)	Isoenergetic CRHP vs. CD (fully provided)	12 weeks (6 per arm)	PP	MRI
Dikariyanto et al.[45] (2020)	UK	Parallel-group RCT	107 adults at above-average cardiovascular risk (50 underwent MRI/MRS)	Almonds (20% energy) vs. control snacks (isoenergetic)	6 weeks	PP	MRI
Thomsen et al.[46] (2022)	Denmark	Parallel-group RCT	72 adults with T2DM (67 completed)	Hypocaloric CRHP vs. CD (matched ~ 6% WL; meals provided)	6 weeks	PP	MRI
Della Pepa et al.[47] (2022)	Italy	Parallel-group RCT	39 adults with T2DM	Multifactorial vs. MUFA diet (no calorie restriction)	8 weeks	PP	MRI
Dokpuang et al.[48] (2023)	New Zealand	Parallel-group RCT	33 adults with obesity and prediabetes (24 completed)	5:2 IF + probiotic vs. IF + placebo	12 weeks	PP	MRI
Sandby et al.[57] (2024)	Denmark	Parallel-group RCT	100 males with abdominal obesity (80 completed; IPFD data available in 73)	400 g/day whole milk vs. yogurt vs. heat-treated yogurt vs. acidified milk	16 weeks	PP	MRI
Harvie et al.[49] (2025)	UK	Parallel-group RCT	20 premenopausal women with obesity	IER (5:2) vs. daily CER; energy-matched	8 weeks	PP	¹H-MRS
Liu Z et al.[26] (2025)	China	Prospective parallel-group interventional study (non-randomized)	41 adults with T2DM (33 completed)	LCFD vs. LCRFD	13 weeks	PP	MRI
Exercise-only interventions							
Langleite et al.[50] (2016)	Norway	Prospective interventional study	22 sedentary adults (dysglycemic vs. normoglycemic)	Endurance + strength (4 sessions/wk)	12 weeks	PP	¹H-MRS
Heiskanen et al.[51] (2018)	Finland	Parallel-group RCT	54 sedentary adults (33 completed)	SIT vs. MICT cycling	2 weeks	ITT	¹H-MRS
Fortuin-de Smidt et al.[52] (2020)	South Africa	Parallel-group RCT	43 adults with obesity (35 completed)	Supervised aerobic exercise + resistance vs. no exercise	12 weeks	PP	MRI
Li et al.[25] (2022)	China	Parallel-group RCT	106 adults with T2DM	Supervised aerobic exercise vs. no exercise	26 weeks	ITT	MRI
Rees et al.[56] (2025)	Canada	Parallel-group RCT	20 adults with T2DM (16 completed; IPFD data available in 15)	Fasted vs. postprandial morning walking exercise	16 weeks	PP	MRI
Combined diet and exercise interventions							
Vogt et al.[53] (2016)	Germany	Prospective interventional study	29 adults with obesity and T2DM	15-w program: formula diet → refeed → maintenance + weekly exercise	15 weeks	PP	MRI
Zhang et al.[58] (2024)	China	Parallel-group RCT	53 adults with overweight/obesity and T2DM; IPFD data available in 39	Control arm only: calorie restricted diet + exercise; sham acupuncture used as control procedure	5 weeks	PP	MRI
Liu H et al.[27] (2025)	China	Prospective interventional study	104 adults with obesity	Caloric restriction + exercise targets; remote monitoring	26 weeks	PP	MRI
Cheng et al.[54] (2026)	China	Prospective interventional study	23 adults with obesity	Calorie restricted diet + structured exercise/lifestyle program	24 weeks	PP	MRI
Other combined lifestyle interventions							
Cadenas-Sanchez et al.[55](2022)	Spain	Prospective parallel-group interventional study (non-randomized)	101 children with overweight/obesity (98 completed)	Aerobic + resistance MVPA program + psychoeducation vs. psychoeducation alone	20 weeks	PP, ITT	MRI

Abbreviations: *CD* conventional diabetes diet, *CER* continuous energy restriction, *CHO* carbohydrate, *CRHP* carbohydrate-reduced high-protein, *IER* intermittent energy restriction, *IF* intermittent fasting, *IPFD* intrapancreatic fat deposition, *ITT* intention-to-treat, *LCFD* low-calorie formula diet, *LCRFD* low-calorie real food-based diet, *MICT* moderate-intensity continuous training, *MRI* magnetic resonance imaging, *MRS* magnetic resonance spectroscopy, *MUFA* monounsaturated fatty acid, *MVPA* moderate-to-vigorous physical activity, *PP* per-protocol, *RCT* randomized controlled trial, *SIT* sprint interval training, *T2DM* type 2 diabetes mellitus, *VLCD* very-low-calorie diet, *WL* weight loss

### Overall Effect of Lifestyle Modification on IPFD

**Fig. 2:**
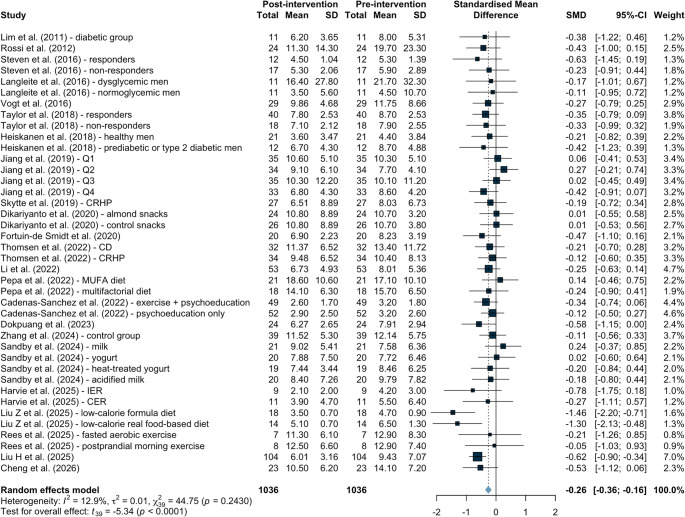
Overall changes in intrapancreatic fat deposition following lifestyle modification. CD, conventional diabetes diet; CER, continuous energy restriction; CRHP, carbohydrate-reduced high-protein; IER, intermittent energy restriction; MUFA, monounsaturated fatty acid; SMD, standardized mean difference; CI, confidence interval. Footnotes: Post-intervention measurements were obtained at the follow-up visit closest to completion of the intervention; subsequent weight maintenance support was not considered part of the intervention. In the study by Zhang et al. [58] only the control arm, which received calorie restriction, exercise, and sham acupuncture, was included in the synthesis; the active acupuncture arm was excluded

**Fig. 3:**
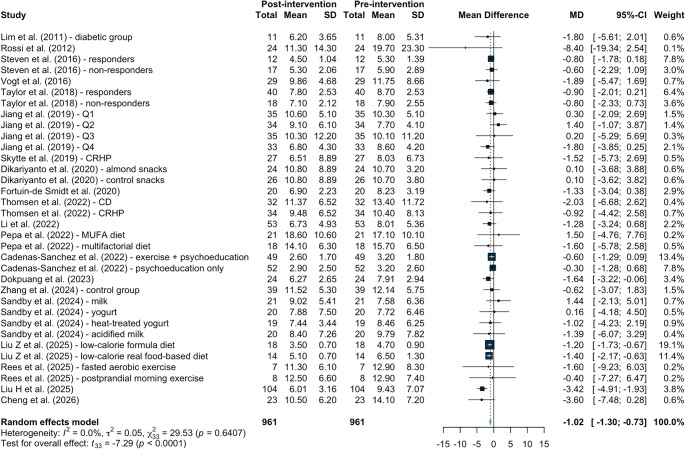
Absolute changes in intrapancreatic fat deposition following lifestyle modification. CD, conventional diabetes diet; CER, continuous energy restriction; CRHP, carbohydrate-reduced high-protein; IER, intermittent energy restriction; MUFA, monounsaturated fatty acid; SMD, standardized mean difference; CI, confidence interval. Footnote: In the study by Zhang et al. [58] only the control arm, which received calorie restriction, exercise, and sham acupuncture, was included in the synthesis; the active acupuncture arm was excluded

**Fig. 4:**
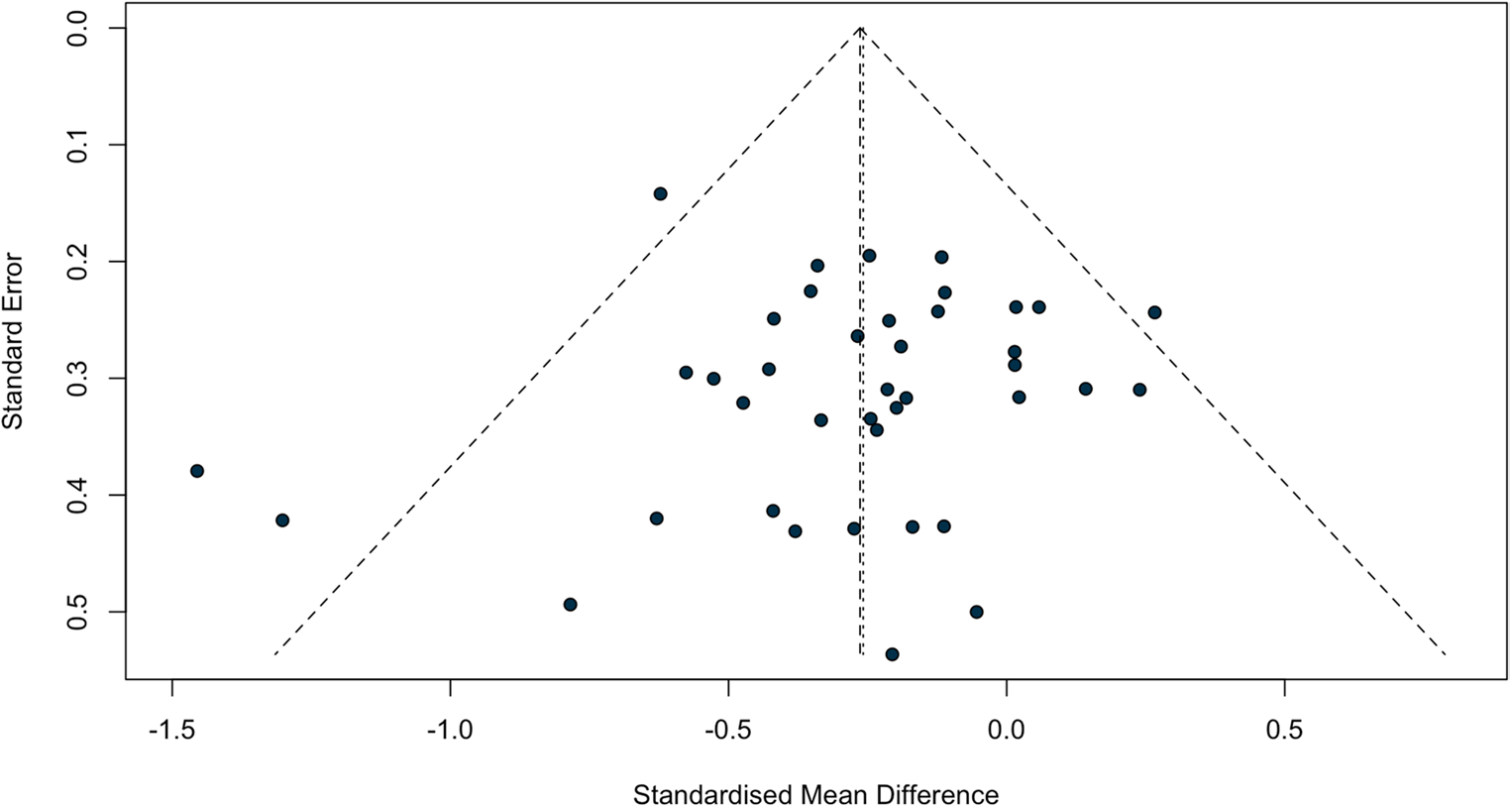
Funnel plot assessing potential publication bias across the included studies

Twenty-three studies, comprising 1036 participants, were included in the meta-analysis. The pooled estimate showed a statistically significant reduction in IPFD (SMD − 0.26, 95% CI -0.36 to -0.16; *p* < 0.0001) following lifestyle modification, with low between-study heterogeneity (I^2^ = 12.9%, τ^2^ = 0.01) (Fig. 2). When expressed as absolute change in IPFD, the pooled MD was − 1.02% (95% CI -1.30 to -0.73) in MRI-based studies, with no evidence of heterogeneity (I^2^ = 0%, τ^2^ = 0.05; *p* < 0.0001) (Fig. 3). Visual inspection of the funnel plot revealed symmetry (Fig. 4), and Egger’s regression test indicated no significant asymmetry (*p* = 0.78).

### Effects of Different Intervention Types on IPFD

The subgroup analysis by intervention type demonstrated consistent reductions in IPFD across all categories (Table 2). Diet-only interventions were associated with a significant reduction in IPFD (SMD − 0.22, 95% CI -0.37 to -0.08), with low between-study heterogeneity (I^2^ = 27.9%, τ^2^ = 0.02). Further stratification of dietary interventions showed a greater reduction following energy-restricted diets (SMD − 0.34, 95% CI -0.56 to -0.13; I^2^ = 42.8%, τ^2^ = 0.06) than after isocaloric/weight-maintenance dietary interventions (SMD − 0.04, 95% CI -0.17 to 0.09; I^2^ = 0%, τ^2^ = 0), with a significant difference between dietary subgroups (*p* = 0.008; Figure S1). Exercise-only interventions yielded a similar magnitude of effect, with a pooled SMD of -0.26 (95% CI -0.36 to -0.15) and no observed heterogeneity (I^2^ = 0%, τ^2^ = 0). The largest numerical effect size was observed for combined diet and exercise interventions, which were associated with a pooled SMD of -0.41 (95% CI -0.81 to -0.02), accompanied by low heterogeneity (I^2^ = 29.4%, τ^2^ = 0.03). Despite numerical differences in effect sizes across intervention categories, formal tests for subgroup differences indicated no statistically significant effect modification between intervention types (*p* = 0.40).

**Table 2:** Subgroup and sensitivity analyses of changes in intrapancreatic fat deposition following lifestyle modification

	Studies, *n*	Participants, *n*	SMD	95% confidence interval	I^2^, %	τ^2^	Subgroup difference
By intervention type^*^							
Diet alone	13	597	-0.22	-0.37 to -0.08	27.9	0.02	0.40
Exercise alone	5	143	-0.26	-0.36 to -0.15	0	0	
Diet and exercise combined	4	195	-0.41	-0.81 to -0.02	29.4	0.03	
By duration of intervention^†^							
< 13 weeks	14	546	-0.15	-0.25 to -0.06	0	0	0.11
13 to < 26 weeks	8	362	-0.32	-0.53 to -0.11	34	0.02	
≥ 26 weeks	2	157	-0.46	-2.82 to 1.91^#^	58.7	0.04	
By method of IPFD measurement							
MRI	20	961	-0.25	-0.36 to -0.14	23.8	0.02	0.67
MRS	3	75	-0.30	-0.52 to -0.07	0	0	
Sensitivity analyses							
Limited to prediabetes/T2DM	14	445	-0.31	-0.46 to -0.16	8.0	< 0.01	-
Limited to overweight/obese	17	819	-0.24	-0.33 to -0.14	0	0.01	-
Limited to low/moderate risk of bias	19	961	-0.24	-0.35 to -0.13	20.4	0.02	-

^*^One study involving a behavioral/psychoeducational intervention was classified as an other combined lifestyle intervention and excluded from intervention-type analyses [55]

^†^ One study reported IPFD at multiple follow-up time points[53], with each time point assigned to the corresponding intervention-duration subgroup

^#^ This estimate should be interpreted with caution because the wide confidence interval is largely attributable to the Hartung–Knapp adjustment, which produces conservative intervals when few studies (two in this case) are included in a meta-analysis

Abbreviations: *SMD*, standardized mean difference, *MRI*, magnetic resonance imaging, *MRS*, magnetic resonance spectroscopy, *T2DM*, type 2 diabetes mellitus

Complementary analyses of absolute changes in MRI-measured IPFD yielded results consistent with the primary SMD-based findings (Table S3). The pooled MD was − 1.04% (95% CI − 1.31 to − 0.78; I^2^ = 0%, τ^2^ = 0) for diet-only interventions, − 1.29% (95% CI − 1.60 to − 0.97; I^2^ = 0%, τ^2^ = 0) for exercise-only interventions, and − 2.46% (95% CI − 4.74 to − 0.19; I^2^ = 26.1%, τ^2^ = 0.90) for combined diet-and-exercise interventions. Although combined interventions showed the greatest numerical reduction in IPFD, the test for subgroup differences did not reach the conventional level of statistical significance (*p* = 0.068).

### Effects of Other Factors on IPFD

No significant differences in the change in IPFD were observed when the effects of lifestyle modification were stratified by either intervention duration (*p* = 0.11) or IPFD assessment method (*p* = 0.67) (Table 2).

When restricted to studies including participants with prediabetes or T2DM, the pooled SMD for IPFD reduction was − 0.31 (95% CI − 0.46 to − 0.16; I^2^ = 8.0%; τ^2^ < 0.01; *p* = 0.0003) (Figure S2). Among studies focusing on overweight or obese populations, the effect remained significant, with a pooled SMD of − 0.24 (95% CI − 0.33 to − 0.14; I^2^ = 0%; τ^2^ = 0.01; *p* < 0.0001) (Figure S3). Furthermore, a sensitivity analysis excluding studies judged to have a high risk of bias yielded consistent results (SMD − 0.24, 95% CI -0.35 to − 0.13; I^2^ = 20.4%; τ^2^ = 0.02; *p* < 0.0001) (Figure S4). Leave-one-out sensitivity analyses demonstrated that the overall reduction in IPFD remained statistically significant following the sequential omission of each study, with pooled estimates ranging from SMD − 0.22 to − 0.30 and consistently low heterogeneity (I^2^ = 0–17.0%) (Table S4).

In univariable meta-regression analyses (Table 3), no significant associations were observed between the SMD in IPFD and the proportion of female participants (*p* = 0.990), baseline BW (*p* = 0.051), baseline IPFD (*p* = 0.325), or SMD in BMI (*p* = 0.142). By contrast, greater % weight loss was significantly associated with larger reductions in IPFD (coefficient = − 0.029, 95% CI − 0.049 to − 0.009; *p* = 0.008), explaining 49.63% of between-study heterogeneity. SMD in LFC was also significantly associated with IPFD SMD in univariable analysis (coefficient = 0.204, 95% CI 0.038 to 0.369; *p* = 0.018), explaining 34.98% of heterogeneity. In a multivariable model, % weight loss and SMD LFC explained 42.09% of heterogeneity, but neither % weight loss (*p* = 0.167) nor SMD LFC (*p* = 0.507) remained independently associated with IPFD change (Table 3).

**Table 3:** Impact of study-level covariates on changes in intrapancreatic fat deposition

	k^*^	*R* ^2^		Estimate	lower CI	upper CI	*p*-value
Univariable meta-regression							
Female, %	26	0%	Intercept	-0.3315	-0.7484	0.0855	0.1139
			Women, %	0.0000	-0.0073	0.0074	0.9898
Baseline BW	27	40.48%	Intercept	-2.0603	-3.8301	-0.2906	0.0243
			Baseline BW	0.0185	-0.0001	0.0371	0.0506
Baseline IPFD	40	0%	Intercept	-0.3745	-0.6308	-0.1182	0.0053
			Baseline IPFD	0.0126	-0.0130	0.0382	0.3245
% Weight loss	23	49.63%	Intercept	-0.0703	-0.2458	0.1051	0.4140
			% Weight loss	-0.0289	-0.0492	-0.0085	0.0077
SMD in BMI	14	0%	Intercept	-0.2749	-0.4861	-0.0637	0.0150
			SMD BMI	0.1835	-0.0708	0.4378	0.1419
SMD in LFC	25	34.98%	Intercept	-0.1225	-0.2767	0.0316	0.1137
			SMD LFC	0.2037	0.0384	0.3690	0.0179
Multivariable meta-regression							
% Weight loss and SMD in LFC	21	42.09%	Intercept	-0.0898	-0.2886	0.1091	0.3554
			% Weight loss	-0.0499	-0.1226	0.0228	0.1666
			SMD LFC	-0.2044	-0.8388	0.4301	0.5071

*k indicates the number of effect estimates included in each model; because some studies contributed more than one observation, k may exceed the number of unique studies

Abbreviations: R^2,^ proportion of between-study heterogeneity explained by the moderator, *CI* confidence interval, *SMD* standardized mean difference, *BW* body weight, *BMI* body mass index, *LFC* liver fat content

## Discussion

This field-wide systematic review and meta-analysis provides the first comprehensive synthesis of published evidence on the effects of lifestyle modification on IPFD. The 23 included interventional studies all featured prospective data collection and quantified IPFD using magnetic resonance–based techniques. The overall pooled analysis demonstrated a significant reduction in IPFD (SMD − 0.26). Moreover, significant reductions in IPFD were observed across diet-only, exercise-only, and combined diet–exercise approaches. Notably, combined diet–exercise interventions yielded the greatest numerical IPFD decrease.

The first global expert consensus on IPFD (the Melbourne Consensus) recognized the modifiability of IPFD as a defining characteristic of this fat depot[2], based on evidence synthesized in recent meta-analyses of glucose-lowering medications and bariatric surgery.[59, 60] Our findings extend this evidence by demonstrating that IPFD is also responsive to lifestyle modification. The absolute reduction observed in this study (-1.0%) is smaller than that reported with glucose-lowering medications (-1.5%) or bariatric surgery (-3.9%).[59, 60] Although modest, these changes are broadly scalable and applicable to the general population, unlike medications and surgery, which have more restricted indications. Lifestyle modification may therefore serve as the first-line strategy for reducing IPFD and as an adjunct to pharmacological or surgical approaches within a holistic management framework. Our analysis further indicates that diet and exercise are similarly effective, with their combination providing the greatest potential benefit. These findings highlight that even basic, non-invasive, and readily available interventions can meaningfully reduce IPFD. They also underscore the importance of incorporating lifestyle strategies early in the management of excessive IPFD to reduce the need for more costly and invasive approaches. Additionally, an important question is whether the observed reduction in IPFD reflects a pancreas-specific response or instead forms part of a broader mobilization of ectopic fat during lifestyle-induced metabolic improvement. Our meta-regression provides some support for the latter possibility. Greater weight loss and reductions in liver fat were associated with larger decreases in IPFD in univariable analyses; however, these associations did not remain significant in the multivariable model and should therefore be interpreted as hypothesis-generating. Collectively, these findings suggest that IPFD may respond, at least in part, to broader changes in energy balance and ectopic fat remodeling rather than representing an entirely pancreas-specific phenomenon.

Our findings reinforce the notion, as highlighted in the PANDORA hypothesis, that IPFD represents a modifiable therapeutic target.[3] The PANDORA hypothesis postulates that reducing IPFD may attenuate pancreatic lipotoxicity and, in turn, improve β-cell function and reduce insulin resistance—mechanisms that could contribute to better glycemic control and lower the risk of complications in individuals with, or at risk for, T2DM.[3] Given the well-established cardiometabolic benefits of lifestyle modification, it is unsurprising that it also exerts beneficial effects on IPFD. However, the potential benefits extend beyond T2DM. Emerging evidence links excessive IPFD with major diseases of the exocrine pancreas[9, 11, 16, 61, 62], suggesting that reductions in IPFD through lifestyle interventions could decrease pancreatic vulnerability to inflammation or neoplastic processes. This highlights a potential role for lifestyle modification as a preventive strategy in patients with a history of pancreatitis and/or risk factors for pancreatic cancer. Collectively, these considerations provide a biologically plausible rationale for the potential benefits of lifestyle modification on exocrine pancreatic outcomes through reduction of IPFD. From a clinical perspective, our findings suggest that the benefits of lifestyle modifications extend to fat in the pancreas, in addition to established effects on fat depots in the liver and skeletal muscle.[1] The observed reduction in IPFD further underscores the importance of lifestyle interventions in individuals with metabolic syndrome.[21, 22] More broadly, these considerations support the inclusion of IPFD within the ectopic fat framework that links obesity, T2DM, and MASLD across the cardiometabolic continuum.

To translate this paradigm shift into clinical practice, several unmet needs must be addressed. There is a clear need for rigorous, adequately powered RCTs designed to detect changes in IPFD. Ideally, such trials should include active control arms to strengthen causal inference and establish the relative efficacy of alternative interventions. Refining lifestyle strategies will require defining dose-response relationships, identifying clinically meaningful thresholds of IPFD reduction, and determining whether specific patient subgroups derive greater benefit (and, hence, supporting personalized prescriptions). Longer follow-up periods are also essential to determine whether IPFD reduction is sustained and to clarify its impact on cardiometabolic health, progression of pancreatic disease, and hard outcomes such as cardiovascular events and cause-specific or all-cause mortality. Last, evaluating whether lifestyle modification exerts additive or synergistic effects when combined with pharmacological therapies may inform more intensive and targeted strategies for the prevention and treatment of diseases of the endocrine pancreas and exocrine pancreas alike.[63, 64].

This study has several limitations that warrant further consideration. First, most of the included studies were not RCTs, and some lacked a comparator group, limiting causal inference. However, all included studies were prospective and interventional in nature, and the consistent direction of IPFD change across diverse study designs supports the robustness of the overall findings. Second, IPFD change was an exploratory endpoint in most studies, and these trials may therefore have been underpowered to detect small effects. Nevertheless, a statistically significant pooled effect was observed, suggesting that lifestyle-induced changes in IPFD are detectable even in the absence of IPFD-focused trial designs. Third, despite extensive subgroup, sensitivity, and meta-regression analyses, certain study-level factors could not be fully accounted for in our analyses, including intervention intensity, adherence, and background medication use. On the other hand, this variability reflects real-world lifestyle interventions across a broad range of clinical contexts, which arguably strengthens the external validity of our findings. Fourth, the predefined exclusion of non–magnetic resonance–based methods for IPFD assessment may have reduced the number of eligible studies; however, this approach ensured methodological consistency and high measurement validity across studies.[65] Last, although Egger’s test did not suggest funnel plot asymmetry, the limited power of the test means that publication bias cannot be fully ruled out. However, the low heterogeneity observed across studies lends greater confidence to the overall findings.

## Conclusion

This systematic review and meta-analysis demonstrates that lifestyle modification—through diet, exercise, or combined approaches—can significantly reduce IPFD. Our findings highlight the modifiability of IPFD and position lifestyle modification as both a preventive strategy and a complementary modality to other approaches for reducing the burden of excessive IPFD. Further well-designed, adequately powered trials are warranted to identify clinically meaningful thresholds of IPFD reduction and to clarify the long-term impact of IPFD reduction on cardiometabolic health and beyond.

## Key References

- Petrov MS, Taylor R. Intra-pancreatic fat deposition: bringing hidden fat to the fore. Nat Rev Gastroenterol Hepatol. 2022;19(3):153-168.
  - The review highlighted IPFD as an important but understudied ectopic fat depot linked to metabolic and pancreatic diseases, while emphasizing major gaps in its definition, measurement, and pathophysiological significance.
- Petrov MS, Yamazaki H, Lu G, et al. Conceptual framework and expert guidance on intrapancreatic fat deposition: the Melbourne consensus. Nat Rev Gastroenterol Hepatol. 2026;Epub ahead of print.
  - The Melbourne consensus establishes fatty pancreas disorder as a measurable, potentially reversible condition associated with pancreatitis, pancreatic cancer, and type 2 diabetes, providing the first unified framework for IPFD research and clinical practice.
- Petrov MS. Fatty change of the pancreas: the Pandora’s box of pancreatology. Lancet Gastroenterol Hepatol. 2023;8(7):671-682.
  - The PANDORA hypothesis positions excessive IPFD as a central determinant of major pancreatic diseases, with potential repercussions that may reshape how these conditions are understood, diagnosed, and managed.
- Souza M, Silva GP, Junior CRO, Amaral MJM, Lima LC V., Charatcharoenwitthaya P. Prevalence, clinical characteristics, and outcomes of fatty pancreas disease: an updated systematic review and meta-analysis. Eur J Gastroenterol Hepatol. 2025;37(2):137-146.
  - This systematic review and meta-analysis of nearly 100,000 individuals showed that fatty pancreas disorder affects one in five individuals and is associated with the development of adverse metabolic and pancreatic outcomes, supporting its emerging clinical relevance.
- Della Pepa G, Salamone D, Testa R, Bozzetto L, Costabile G. Intrapancreatic fat deposition and nutritional treatment: the role of various dietary approaches. Nutr Rev. 2024;82(12):1820-1834.
  - This narrative review summarizes the evidence supporting the use of dietary interventions to reduce IPFD, emphasizing nutrition as a plausible strategy for mitigating associated metabolic risks.
- Wang Y, Liu Y, Petrov MS. The effects of metabolic bariatric surgery on intra-pancreatic fat deposition and total pancreas volume: a systematic review and meta-analysis. Obes Surg. 2025;35(4):1513-1524.
  - This meta-analysis showed that metabolic bariatric surgery substantially reduces IPFD and may also influence total pancreas volume.
- Agon HC, Shen Y, Petrov MS. Effects of glucose‐lowering medications on intrapancreatic fat deposition: a systematic review and meta‐analysis of randomized controlled trials. Obes Rev. 2026;27(6):e70087.
  - This synthesis of randomized controlled trials showed that glucose-lowering medications can reduce IPFD, supporting IPFD as a modifiable ectopic fat depot and potential treatment-related biomarker in diabetes.

## Supplementary Information

Below is the link to the electronic supplementary material.

Supplementary Material 1

## Data Availability

All data generated or analyzed during this study are included in this published article and its supplementary information files.

## Abbreviations

IPFD: intrapancreatic fat deposition
MRI: magnetic resonance imaging
MRS: magnetic resonance spectroscopy
T2DM: type 2 diabetes mellitus
MASLD: metabolic dysfunction-associated steatotic liver disease
BW: body weight
BMI: body mass index
LFC: liver fat content
SMD: standardized mean difference
MD: mean difference
SD: standard deviation
RCT: randomized controlled trial
PRISMA: Preferred Reporting Items for Systematic Reviews and Meta-Analyses
MOOSE: Meta-analysis of Observational Studies in Epidemiology
ROB 2: Cochrane Risk of Bias 2 tool
ROBINS-I: Risk of Bias in Non-randomized Studies of Interventions
