# Supplementary material for: Effects of Lifestyle Modification on Intrapancreatic Fat Deposition: A Systematic Review and Meta-Analysis
Source: Curr Obes Rep. 2026 Jul 20;15(1):60. doi: 10.1007/s13679-026-00737-0 (PMC13384985; doi:10.1007/s13679-026-00737-0)
Supplement: Supplementary file 1 — Supplementary Material 1 [file 13679_2026_737_MOESM1_ESM.docx]

**SUPPLEMENTARY MATERIAL**

**Effects of Lifestyle Modification on Intrapancreatic Fat Deposition: A Systematic Review and Meta-analysis**

**Summary of contents**

SUPPLEMENTARY METHODS2

SUPPLEMENTARY FIGURES4

SUPPLEMENTARY TABLES8

SUPPLEMENTARY REFERENCES18

**SUPPLEMENTARY METHODS**

***Search strategy***

We searched the (A) PubMed and (B) Embase databases (from inception to February 25, 2026) using the following search strategy:

1. **PubMed**

( "fatty pancreas"[tiab] OR "pancreatic fat"[tiab] OR "pancreatic steatosis"[tiab] OR "intrapancreatic fat"[tiab] OR "pancreatic lipomatosis"[tiab] OR "nonalcoholic fatty pancreas disease"[tiab] OR "nonalcoholic fatty pancreas disease"[tiab] OR NAFPD[tiab] OR "pancreatic triacylglycerol"[tiab] OR "pancreas triacylglycerol"[tiab] OR (pancrea*[tiab] AND (fat*[tiab] OR steato*[tiab] OR lipid*[tiab] OR lipomatosis[tiab] OR ectopic[tiab] OR hyperecho*[tiab])) ) AND ( diet[tiab] OR dietary[tiab] OR nutrition[tiab] OR "caloric restriction"[tiab] OR "calorie restriction"[tiab] OR "energy restriction"[tiab] OR "low-calorie"[tiab] OR "very-low-calorie diet"[tiab] OR VLCD[tiab] OR "hypocaloric"[tiab] OR "weight loss"[tiab] OR "weight reduction"[tiab] OR exercise[tiab] OR "physical activity"[tiab] OR training[tiab] OR aerobic[tiab] OR "resistance training"[tiab] OR "strength training"[tiab] OR HIIT[tiab] OR endurance[tiab] OR lifestyle[tiab] OR "behavioral intervention"[tiab] OR "behavior change"[tiab] ) AND ( "magnetic resonance"[tiab] OR MRI[tiab] OR MRS[tiab] OR "magnetic resonance spectroscopy"[tiab] OR "chemical shift"[tiab] OR "pancreatic fat content"[tiab] OR "chemicalshift"[ tiab] OR Dixon[tiab] OR IDEAL[tiab] OR "fat fraction"[tiab] OR PDFF[tiab] OR "proton density fat fraction"[tiab] OR "chemical shift encoded"[tiab] )

1. **Embase**

( 'fatty pancreas':ti,ab OR 'pancreatic fat':ti,ab OR 'pancreatic steatosis':ti,ab OR 'intrapancreatic fat':ti,ab OR 'pancreatic lipomatosis':ti,ab OR 'nonalcoholic fatty pancreas disease':ti,ab OR 'nonalcoholic fatty pancreas disease':ti,ab OR nafpd:ti,ab OR 'pancreatic triacylglycerol':ti,ab OR 'pancreas triacylglycerol':ti,ab OR (pancrea*:ti,ab AND (fat*:ti,ab OR steato*:ti,ab OR lipid*:ti,ab OR lipomatosis:ti,ab OR ectopic:ti,ab OR hyperecho*:ti,ab)) ) AND ( diet:ti,ab OR dietary:ti,ab OR nutrition:ti,ab OR 'caloric restriction':ti,ab OR 'calorie restriction':ti,ab OR 'energy restriction':ti,ab OR 'low-calorie':ti,ab OR 'very-low-calorie diet':ti,ab OR vlcd:ti,ab OR hypocaloric:ti,ab OR 'weight loss':ti,ab OR 'weight reduction':ti,ab OR exercise:ti,ab OR 'physical activity':ti,ab OR training:ti,ab OR aerobic:ti,ab OR 'resistance training':ti,ab OR 'strength training':ti,ab OR hiit:ti,ab OR endurance:ti,ab OR lifestyle:ti,ab OR 'behavioral intervention':ti,ab OR 'behavior change':ti,ab ) AND ( 'magnetic resonance':ti,ab OR mri:ti,ab OR mrs:ti,ab OR 'magnetic resonance spectroscopy':ti,ab OR 'chemical shift':ti,ab OR 'chemical-shift':ti,ab OR 'pancreatic fat content':ti,ab OR dixon:ti,ab OR ideal:ti,ab OR 'fat fraction':ti,ab OR pdff:ti,ab OR 'proton density fat fraction':ti,ab OR 'chemical shift encoded':ti,ab)

**SUPPLEMENTARY** **FIGURES**

**Figure S1.** Stratified analysis by type of dietary intervention.

**
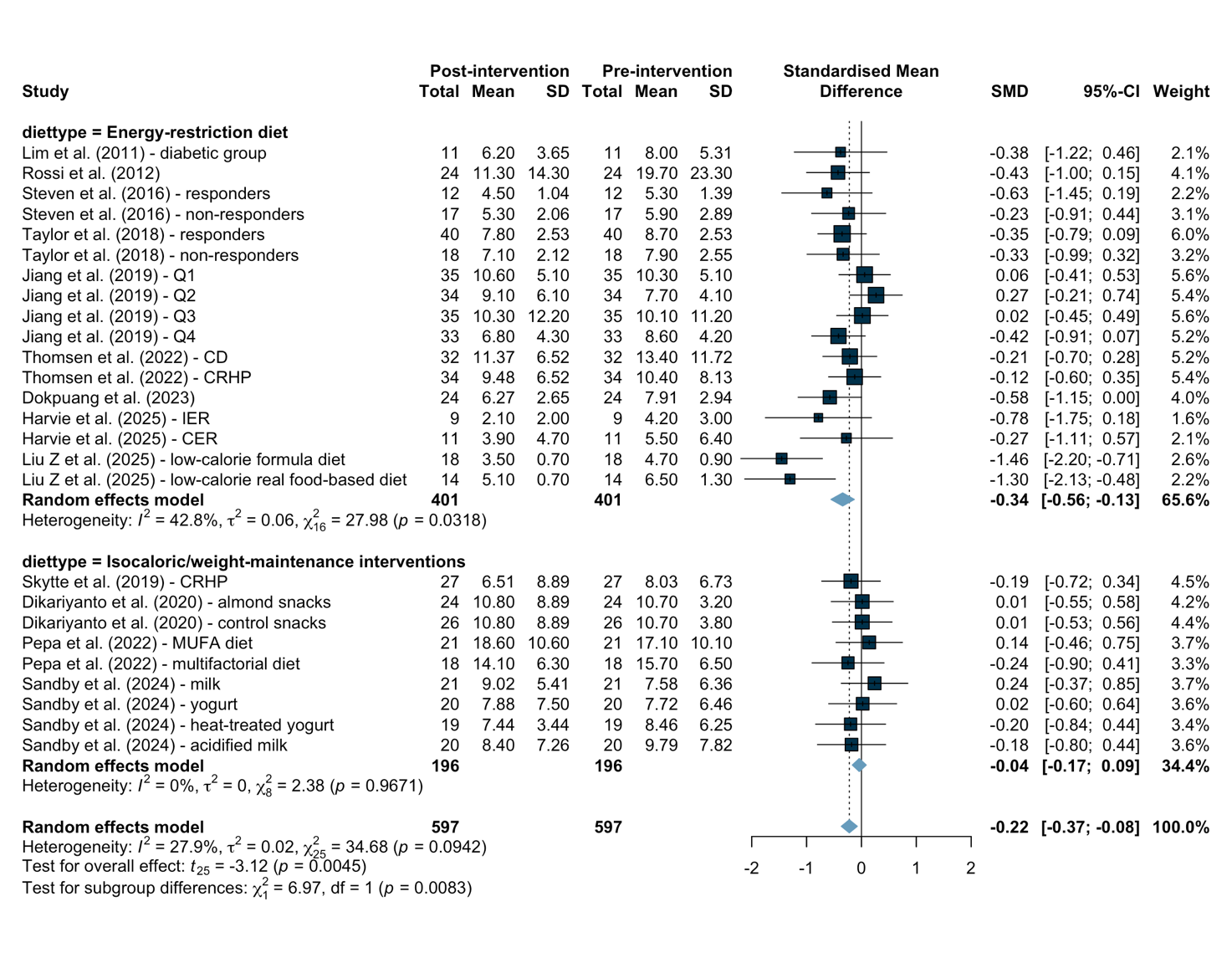
**

**Figure S2.** Sensitivity analysis of studies conducted in prediabetes/type 2 diabetes mellitus populations.


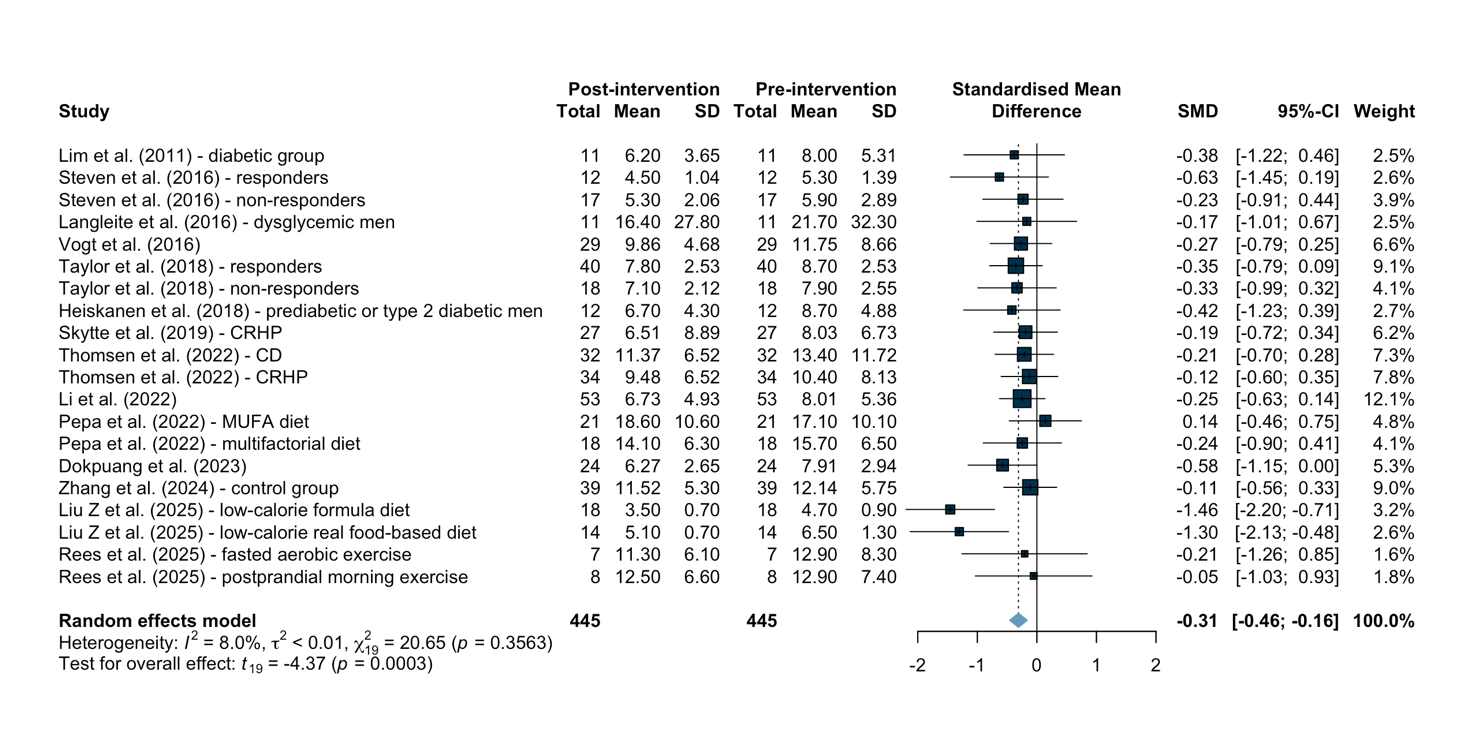


**Figure S3.** Sensitivity analysis of studies conducted in overweight/obese populations.


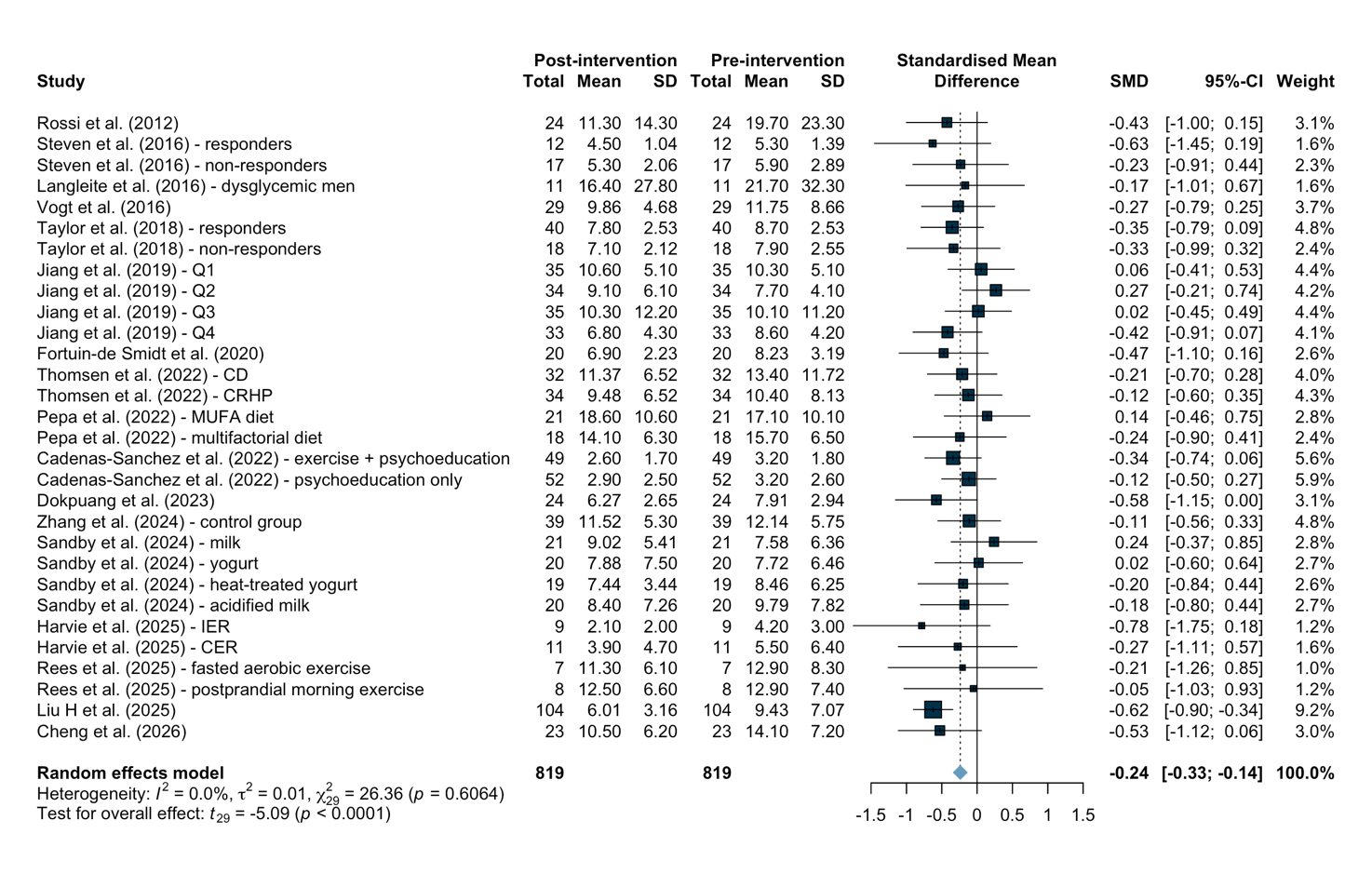


**Figure S4.** Sensitivity analysis of studies with low-to-moderate risk of bias.

**
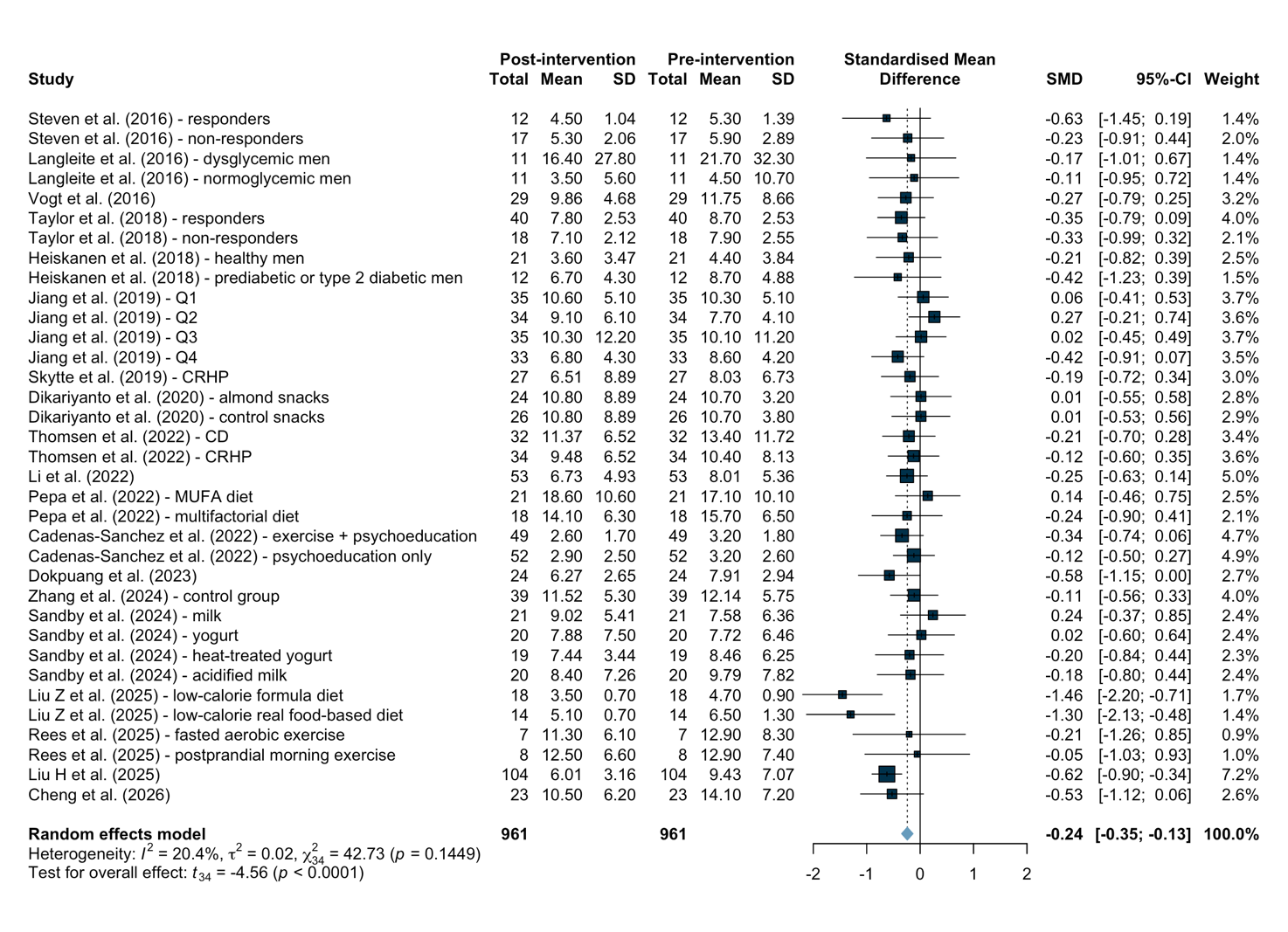
**

**SUPPLEMENTARY TABLES**

**Table S1.** Risk of bias assessment of the included studies.

1. RCT

| **First author (year)** | **Domain 1** | **Domain 2** | **Domain 3** | **Domain 4** | **Domain 5** | **Overall** |
| --- | --- | --- | --- | --- | --- | --- |
|  | **Bias arising from the randomization process** | **Bias due to deviations from the intended interventions (effect of assignment to intervention)** | **Bias due to Missing outcome data** | **Bias in measurement of the outcome** | **Bias in selection of the reported result** |  |
| Steven et al.^1^ (2016) | Some concerns | Some concerns | Low | Low | Low | Some concerns |
| Taylor et al.^2^ (2018) | Some concerns | Some concerns | Low | Low | Low | Some concerns |
| Heiskanen et al.^3^ (2018) | Low | Some concerns | Some concerns | Some concerns | Low | Some concerns |
| Skytte et al.^4^ (2019) | Low | Some concerns | Low | Low | Low | Some concerns |
| Dikariyanto et al.^5^ (2020) | Low | Some concerns | Low | Low | Low | Some concerns |
| Fortuin-de Smidt et al.^6^ (2020) | Low | Some concerns | High | Low | Some concerns | High |
| Thomsen et al.^7^ (2022) | Low | Some concerns | Low | Low | Some concerns | Some concerns |
| Li et al.^8^ (2022) | Low | Some concerns | Low | Low | Some concerns | Some concerns |
| Pepa et al.^9^ (2022) | Some concerns | Some concerns | Some concerns | Low | Low | Some concerns |
| Dokpuang et al.^10^ (2023) | Low | Low | Some concerns | Low | Low | Some concerns |
| Zhang et al.^11^ (2024) | Low | Some concerns | Some concerns | Low | Low | Some concerns |
| Sandby et al.^12^ (2024) | Low | Low | Some concerns | Low | Low | Some concerns |
| Harvie et al.^13^ (2025) | Low | Some concerns | High | Some concerns | Some concerns | High |
| Rees et al.^14^ (2025) | Low | Some concerns | Some concerns | Low | Low | Some concerns |

1. Non-RCT

| **First author (year)** | **Domain 1** | **Domain 2** | **Domain 3** | **Domain 4** | **Domain 5** | **Domain 6** | **Overall** |
| --- | --- | --- | --- | --- | --- | --- | --- |
|  | **Bias due to confounding** | **Bias in classification of interventions** | **Bias in selection of participants into the study (or into the analysis)** | **Bias due to missing data** | **Bias in measurement of the outcome** | **Bias in selection of the reported result** |  |
| Lim et al.^15^ (2011) | Moderate | Low | Moderate | Serious | Low | Moderate | Serious |
| Rossi et al.^16^ (2012) | Moderate | Low | Moderate | Critical | Low | Low | Critical |
| Langleite et al.^17^ (2016) | Moderate | Low | Moderate | Low | Moderate | Low | Moderate |
| Vogt et al.^18^ (2016) | Moderate | Low | Low | Low | Low | Moderate | Moderate |
| Jiang et al.^19^ (2019) | Moderate | Low | Low | Low | Low | Moderate | Moderate |
| Cadenas-Sanchez et al.^20^ (2022) | Moderate | Low | Moderate | Low | Low | Low | Moderate |
| Liu Z et al.^21^ (2025) | Moderate | Low | Low | Moderate | Low | Moderate | Moderate |
| Liu H et al.^22^ (2025) | Moderate | Low | Low | Moderate | Low | Low | Moderate |
| Cheng et al.^23^ (2026) | Moderate | Low | Low | Moderate | Low | Low | Moderate |

**Table S2.** Expanded summary of studies examining the effects of lifestyle Interventions on intrapancreatic fat deposition.

| **First author (year)** | **Location** | **Population** | **Intervention(s)** | **Duration** | **Analysis type** | **IPFD measurement** | **Main findings (IPFD-focused)** |
| --- | --- | --- | --- | --- | --- | --- | --- |
| **Diet-only interventions** | | | | | | | |
| Lim et al.^15^ (2011) | UK | 11 adults with T2DM (mean age 49.5y; mean BMI 33.6 kg/m^2^; 18.2% women; diabetes duration <4y) | **Very-low-energy diet** (~600 kcal/day): Optifast liquid formula (510 kcal/day; 46% CHO, 32.5% protein, 20% fat) + 3 servings of non-starchy vegetables | 8 weeks | PP | MRI (3.0T), 3-point Dixon technique; 2 pancreatic ROIs; fat fraction (%) calculated | **At 8 weeks (ΔBW −15%):** IPFD ↓ from 8.0% → 6.2% (Δ−1.8 p.p.; ~22% relative decrease)  *Post hoc*:  **At 12-week follow-up (after resuming normal diet; ΔBW +3.1 kg):** IPFD ↓ further from 6.2% → 5.7% (Δ−0.5 p.p.; ~8% further relative decrease) |
| Rossi et al.^16^ (2012) | Italy | 24 obese adults (mean age 46.7y; mean BMI 35.4 kg/m^2^; 45.8% women) | **Hypocaloric diet:** 500 kcal/day below measured resting energy expenditure × physical activity factor (1.4). Diet composition: 62% CHO, 24% fat, 14% protein, 20 g fiber/day | 13-26 weeks (time to achieve ≥7-10% weight loss) | PP | MRI (1.5T), in-phase/opposed-phase T1-weighted gradient-echo; 3 pancreatic ROIs (head, body, tail); % signal intensity loss | **After ≥7-10% weight loss (ΔBW −8.9%):** IPFD ↓ from 19.7% → 11.3% (Δ−8.4 p.p.; ~42% relative decrease) |
| Steven et al.^1^ (2016) | UK | 30 adults with T2DM (12 responders: mean BMI 34.0 kg/ m^2^ / 17 non-responders: mean BMI 34.4 kg/ m^2^); 29 completed the full protocol | **Very-low-calorie diet:** 624–700 kcal/day (OPTIFAST shakes + non-starchy vegetables)  Diet-focused; physical activity encouraged but not prescribed | 8 weeks | PP | MRI (3.0T), three-point Dixon, two pancreatic ROIs; pancreatic triglyceride fraction (%) averaged across slices; blinded analysis | **At 8 weeks:**  - Responders (ΔBW -15.7%): IPFD ↓ from 5.3% → 4.5% (Δ−0.8 p.p.; ~15.1% relative decrease)  - Non-responders (ΔBW -13.5%): IPFD ↓ from 5.9% → 5.3% (Δ−0.6 p.p.; ~10.2% relative decrease)  **At 6-month (after 2-week stepped return to isocaloric normal foods + 6-month structured weight-maintenance program):**  - Responders (ΔBW +0.4%): IPFD remained stable 4.5%→ 4.4%  - Non-responders (ΔBW +1.4%): IPFD remained stable 5.3%→ 5.0% |
| Taylor et al.^2^ (2018) | UK | 58 adults with T2DM (40 responders: mean age 53.0y; mean BMI 34.9 kg/m^2^; diabetes duration 2.7y / 18 non-responders: mean age 53.3y; mean BMI 35.7 kg/ m^2^; diabetes duration 3.8y) | **Counterweight Plus program**: 825–853 kcal/day formula diet for 12–20 weeks;  All anti-diabetes medications stopped on day 1 | 12–20 weeks (weight-loss phase) | PP | MRI (3T), 3-point Dixon (mDixon); Philips Achieva scanner; pancreatic fat (%) from chemical-shift fat fraction maps | **At 12-20 weeks:**  - Responders (ΔBW -16.2kg): IPFD ↓ from 8.7% → 7.8% (Δ−0.9 p.p.; ~10.3% relative decrease)  - Non-responders (ΔBW -13.4kg): IPFD ↓ from 7.9% → 7.1% (Δ−0.8 p.p.; ~10.1% relative decrease)  No significant difference between-groups  **Up to 12 months (after weight-maintenance phase [2–6 week food reintroduction + structured weight-maintenance support]):**  - Responders (ΔBW +3.3kg), n=29: IPFD remained stable → 7.9%  - Non-responders (ΔBW +4.9kg), n=16: IPFD remained stable → 6.8% |
| Jiang et al.^19^ (2019) | Germany | 137 non-diabetic overweight/obese adults (35–65 y, mean age 50.2; ~52.6% women; mean BMI 31.4 kg/m^2^) | **Calorie-restriction program** (intermittent CR vs continuous CR vs control); analysis pooled arms and stratified by achieved weight loss | 12 weeks | PP | MRI (1.5T), multi-echo GRE PDFF mapping; 3 pancreatic ROIs (head/body/tail), fat fraction (%) | **At 12 weeks:** Only the highest weight loss quartile (Q4) showed substantial IPFD reduction. Q1–Q3 showed no meaningful decrease:  - Q1 (ΔBW 0%): IPFD ↑ from 10.3% → 10.6% (Δ+0.3 p.p.; ~3% relative increase)  - Q2 (ΔBW −3.2%): IPFD ↑ from 7.7% → 9.1% (Δ+1.4 p.p.; ~18% relative increase)  - Q3 (ΔBW −6.1%): IPFD ↑ from 10.1% → 10.3% (Δ+0.2 p.p.; ~2% relative increase)  - Q4 (ΔBW −11.3%): IPFD ↓ from 8.6% → 6.8% (Δ−1.8 p.p.; ~21% relative decrease)  *Post hoc*:  **At 50-week follow-up (after 12-week weight-maintenance phase [structured dietary guidance + no caloric restriction] followed by a 26-week unsupervised follow-up):** Q4 showed further IPFD reduction, while Q1–Q3 had minimal, non-meaningful declines:  - Q1 (ΔBW +1.2%): IPFD ↓ from 10.6% → 10.1%  (Δ−0.5 p.p.; ~5% further relative decrease)  - Q2 (ΔBW -1.3%): IPFD ↓ from 9.1% → 8.5%  (Δ−0.6 p.p.; ~7% further relative decrease)  - Q3 (ΔBW -4.3%): IPFD ↓ from 10.3% → 9.7%  (Δ−0.6 p.p.; ~6% further relative decrease)  - Q4 (ΔBW -11.1%): IPFD ↓ from 6.8% → 6.5%  (Δ−0.3 p.p.; ~4% further relative decrease) |
| Skytte et al.^4^ (2019) | Denmark | 28 adults with T2DM (mean age 64y; mean BMI 30.1 kg/m^2^; diabetes duration 7y; 28.6% women);  27 with valid IPFD measurements | Two isoenergetic diets compared in crossover fashion:  - **CRHP diet:** 30% carbohydrate, 30% protein, 40% fat  - **CD (conventional diabetes) diet:** 50% carbohydrate, 17% protein, 33% fat  Both diets were fully provided, designed to maintain weight stability | 6 weeks per arm (total 12 weeks) | PP | MRI (3.0T), Philips Ingenia; using chemical-shift water–fat imaging (mDixon); duplicate pancreatic fat fraction (%), averaged | **At 6 weeks:**  - CRHP (ΔBW -1.4 kg): IPFD ↓ Δ−1.7 p.p. (median; IQR -3.5 to 0.6)  - CD (ΔBW -0.8 kg): IPFD ↑ Δ+0.5 p.p. (median; IQR -1.0 to 2.0)  Between-diet difference: significant (p<0.05) |
| Dikariyanto et al.^5^ (2020) | UK | 107 adults at above-average cardiovascular risk (56 in almond group: mean age 56.3y; mean BMI 27.3 kg/m^2^; 69.6% women / 51 in control group: mean age 56.0y; mean BMI 26.7 kg/m^2^; 70.6% women); free-living snack consumers; 50 underwent MRI/MRS | **- Almond group:** whole roasted almonds, providing 20% of daily energy requirement  **- Control group:** mini-muffin snacks matching average UK snack macronutrient profile  Both were isoenergetic and intended not to induce weight change | 6 weeks (after 2-week run-in) | PP | MRI (1.5T), Siemens Magnetom Aera; 2-point Dixon sequence; three 1-cm² ROIs (head, body, tail); pancreatic fat fraction (%) = F/(F+W); mean of the three ROIs | **At 6 weeks (ΔBW ≈0 kg):**  - Almonds group: IPFD unchanged Δ+0.1 p.p. (median; IQR -1.1 to 1.4)  - Control group: IPFD unchanged Δ+0.1 p.p. (median; IQR -1.1 to 1.3) |
| Thomsen et al.^7^ (2022) | Denmark | 72 adults with T2DM (mean age 66 y; mean BMI 33 kg/m²; 47.2% women; diabetes duration ~8 years);  67 participants completed the intervention | Two hypocaloric diets inducing matched ~6% weight loss:  - **CRHP diet:** 30%E CHO / 30%E protein / 40%E fat  **- CD diet:** 50%E CHO / 17%E protein / 33%E fat  All meals fully provided | 6 weeks (5 weeks weight loss + 1 week weight stabilization) | PP | MRI (3.0T), Philips Ingenia; chemical-shift encoded water–fat imaging (mDixon); pancreatic fat fraction measured in duplicate, whole pancreas, averaged | **At 6 weeks:**  - CRHP diet (ΔBW -5.8 kg): IPFD ↓ Δ-1.1 p.p. (median; IQR -2.9 to 1.2)  - CD diet (ΔBW -5.8 kg): IPFD ↓ Δ-2.1 p.p. (median; IQR -3.5 to -0.5)  Between-diet difference: significant (p=0.01) |
| Della Pepa et al.^9^ (2022) | Italy | 39 adults with T2DM, abdominal obesity, well-controlled HbA1c ≤7.5%, low physical activity  (21 on MUFA diet: mean age 64y; mean BMI 31 kg/m^2^; 43% women  / 18 on multifactorial diet: mean age 64y; mean BMI 32 kg/m^2^; 44% women) | **- Multifactorial isocaloric diet:** rich in MUFA, PUFA, fiber, polyphenols, vitamins (D/E/C); no calorie restriction  **- MUFA-rich isocaloric diet:** similar macronutrients, lower fiber/PUFA/polyphenols  Both isoenergetic; 800-kcal standardized test meal matched to assigned diet | 8 weeks (after 3-week run-in) | PP | MRI (3T), mDIXON multi-echo PDFF mapping; ROIs in head/body/tail; PDFF (%) from voxelwise mapping | **At 8 weeks:**  - Multifactorial diet (ΔBW -1.5%): IPFD ↓ from 15.7% → 14.1% (Δ−1.6 p.p.; ~10.2% relative decrease, p=0.024)  - MUFA diet (ΔBW -1.2%): IPFD unchanged from 17.1% → 18.6% (Δ+1.5 p.p.; ~8.8% relative increase, p=0.139)  Between-group difference: significant (p=0.014; adjusted p=0.035) |
| Dokpuang et al.^10^ (2023) | New Zealand | 24 obese adults with prediabetes and completed MRI at both timepoints (mean age 54y; mean BMI 34 kg/m^2^; 66.7% women) | **Intermittent fasting 5:2** (600 kcal/day women; 650 kcal/day men on fasting days)  All participants were randomized to receive probiotic or placebo  Dietitian support via counseling + digital tools | 12 weeks | PP | MRI (3T), Siemens Skyra; conventional volumetric pancreatic fat (%); operator-defined segmentation | **At 12 weeks (ΔBW -5%):** IPFD ↓ from 7.70% → 6.54% (Δ−1.16 p.p.; ~15.1% relative decrease, p<0.001)  Probiotic vs placebo: no significant difference (p=0.232) |
| Sandby et al.^12^ (2024) | Denmark | 100 men with abdominal obesity were included (90 initiated intervention -> 80 completed); aged 30–70y, BMI 28.0–45.0 kg/m², waist circumference ≥102 cm; no diabetes, liver, heart, or kidney disease; among those initiating intervention: mean BMI ~32.3–32.7 kg/m² | Four weight-maintenance/eucaloric food-based dairy interventions after 4-week standardization with 400 g/day whole milk:  **- Whole milk:** 400 g/day  **- Whole milk yogurt with live bacteria:** 400 g/day  **- Heat-treated whole milk yogurt with inactivated bacteria:** 400 g/day  **- Chemically acidified whole milk:** 400 g/day  Products were consumed as part of habitual diet; participants were asked to avoid other dairy except ≤1 dL milk and 25 g butter/day | 16 weeks (after 4-week standardization lead-in) | PP | MRI (3.0T), Philips Ingenia/Achieva); chemical shift encoding-based water–fat imaging mDixon | **At 16 weeks:**  IPFD showed no meaningful/significant change overall and no clear between-group differences.  Reported medians:  - Milk: IPFD 6.5% → 8.3% (Δ +1.8 p.p.; ~27.7% relative increase)  - Yogurt: IPFD 6.6% → 6.8% (Δ +0.2 p.p.; ~3.0% relative increase)  - Heat-treated yogurt: IPFD 8.1% → 8.2% (Δ +0.1 p.p.; ~1.2% relative increase)  - Acidified milk: IPFD 9.0% → 7.0% (Δ −2.0 p.p.; ~22.2% relative decrease) |
| Harvie et al.^13^ (2025) | UK | 20 premenopausal women with obesity who completed interventions (mean age 40.1y; mean BMI 34.7 kg/m^2^; 95% White) | **- IER (5:2 intermittent energy restriction):**  - 2 days/week: ~600 kcal/day, very-low–carbohydrate (≤50 g CHO), ~70% energy restriction  - 5 days/week: Mediterranean diet (~1900 kcal/day; 45% CHO, 25% protein, 30% fat)  **- CER (daily 25% energy restriction):** Mediterranean diet ~1500 kcal/day; same macronutrient targets as IER  Both interventions energy-matched on a weekly basis | 8 weeks | PP | ¹H-MRS (1.5T), Philips Achieva; single-voxel water–fat spectroscopy of the whole pancreas; PFF (%) calculated from fat/(fat+water) signal ratio | **At 8 weeks:**  - IER (ΔBW -7.2%): IPFD ↓ from 4.2% → 2.1% (Δ−2.1 p.p.; ~50% relative decrease)  - CER (ΔBW -6.4%): IPFD ↓ from 5.5% → 3.9% (Δ−1.6 p.p.; ~29.1% relative decrease)  Between-diet difference: not significant (p=0.58) |
| Liu Z et al.^21^ (2025) | China | 32 adults with T2DM who completed interventions (18 on LFCD:  mean age 36.5y; 27.8% women / 14 on LCRFD: mean age 44y; 42.9% women) | **- LCFD:** 815–835 kcal/day  **- LCRFD:** 815–835 kcal/day  Anti-diabetic medications stopped on day 1 | 13 weeks (intensive weight loss phase) | PP | MRI IDEAL-IQ (3T), GE Signa Premier; three pancreatic ROIs; PDFF (%) averaged across independent analyses | **At 3 months:**  - LFCD (ΔBW -12.9%): IPFD ↓ from 4.7% → 3.5% (Δ−1.2 p.p.; ~25.5% relative decrease)  - LCRFD (ΔBW -11.8%): IPFD ↓ from 6.5% → 5.1% (Δ−1.4 p.p.; ~21.5% relative decrease)  Between-diet difference: not significant (similar reductions)  **At 6 months (after weight-maintenance phase):**  - LFCD (ΔBW +1.7%): IPFD ↓ from 3.5% → 3.6% (Δ+0.1 p.p.; ~2.9% relative increase)  - LCRFD (ΔBW -0.6%): IPFD ↓ from 5.1% → 4.2% (Δ−0.9 p.p.; ~17.6% relative decrease) |
| **Exercise-only interventions** | | | | | | | |
| Langleite et al.^17^ (2016) | Norway | 22 sedentary men aged 40–65 years (11 dysglycemic overweight: mean age 53y; mean BMI 27.8kg/m^2^ / 11 normoglycemic controls: mean age 53y; mean BMI 23.3kg/m^2^) | **Combined endurance + strength training:** supervised, 4 sessions/week (2 whole-body strength sessions + 2 cycling interval sessions) | 12 weeks | PP | ¹H-MRS (1.5T), Philips Achieva; single-voxel proton spectroscopy; reported as lipid fraction (arbitrary units, AU) derived from fitted lipid and water signal amplitudes | **At 12 weeks:**  - Dysglycemic men (ΔBW -1.2%): IPFD ↓ from 21.7 AU → 16.4 AU (Δ−5.3 AU.; ~24.4% relative decrease)  - Normoglycemic control (ΔBW +0.9%): IPFD ↓ from 4.5 AU → 3.5 AU (Δ−1.0 AU.; ~22.2% relative decrease) |
| Heiskanen et al.^3^ (2018) | Finland | 54 sedentary adults aged 40–55 years (28 healthy men and  26 prediabetic or type 2 diabetic) | Two supervised exercise programs (6 sessions/2 weeks): - **SIT**: sprint interval training (4–6 × 30-sec all-out cycling; 4-min recovery) - **MICT**: moderate-intensity continuous cycling (40–60 min, 60% of peak workload) | 2 weeks | ITT | ¹H-MRS (1.5T), Philips Gyroscan Intera; single-voxel spectroscopy in the pancreatic body; fat (%) determined via standard MRS triacylglycerol quantification | **At 2 weeks:**  - Healthy men: IPFD ↓ from 4.4% (median; IQR 3.0 to 6.1) → 3.6% (median; IQR 2.4 to 5.2) (Δ-0.8 p.p., ~18.2% relative decrease)  - Prediabetes/T2DM men: IPFD ↓ from 8.7% (median; IQR 6.0 to 11.9) → 6.7% (median; IQR 4.4 to 9.6) (Δ-2.0 p.p., ~23% relative decrease)  No difference between exercise modalities |
| Fortuin-de Smidt et al.^6^ (2020) | South Africa | 43 Black South African women with obesity (20 exercise group: median age 22y; mean BMI 34.1kg/m^2^ / 15 control group: median age 23y; mean BMI 33.4kg/m^2^); 35 completed the protocol | **- Exercise group:** supervised combined aerobic (75–80% HRpeak) + resistance training (60–70% HRpeak), 40–60 min/session, 4 days/week  **- Control:** habitual lifestyle | 12 weeks | PP | MRI (3.0T), 3-point Dixon; three 1-cm² ROIs in head, body, tail; pancreatic fat fraction (%) | **At 12 weeks:**  - Exercise group (ΔBW -0.9%): IPFD unchanged from 7.8% (median; IQR 6.4 to 10.4) → 6.9% (median; IQR 5.5 to 8.3) (Δ-0.9 p.p.)  Control also unchanged. No between-group differences |
| Li et al.^8^ (2022) | China | 106 adults with T2DM (mean age 66.4 y; mean BMI 24.5 kg/m^2^; 51% women) | **- Aerobic training group:** supervised moderate-intensity aerobic dancing, 3×/week, each session: 5-min warm-up, 50-min aerobic dance, 5-min cool-down; intensity targeted at 60–70% HRmax. 2-week run-in with progressive intensity  **- Control group:** maintain usual activity; both groups received monthly diabetes self-management education + individualized meal plans (not caloric restriction) | 26 weeks | ITT | MRI (3.0T) using IDEAL-IQ sequence, GE Discovery MR750w; pancreatic regions (head, body, tail) measured 3× each; proton-density fat fraction (PDFF, %) averaged; radiologist blinded to allocation | **At 6 months:**  - Aerobic training: IPFD ↓ from 8.01% → 6.73% (Δ-1.28 p.p., ~16% relative decrease)  - Control: IPFD ↑ from 10.26% → 11.09% (Δ+0.84 p.p., ~8.2% relative increase)  Between-group difference: significant (p=0.001) |
| Rees et al.^14^ (2025) | Canada | 20 adults with T2DM randomized (mean age 59.8y, mean BMI 32.5 kg/m², 50% women); 16 completed; 15 in MRI analysis (FAST n=7, FED n=8) | Morning walking exercise 3×/week, progressing to 180 min/week.  - **FAST:** exercise after overnight fast before breakfast  -**FED:** exercise within 1h after breakfast | 16 weeks | PP | MRI (3T), Siemens PRISMA; multiecho chemical-shift PDFF; pancreatic PDFF (%) from multiple manually selected pancreatic ROIs | **At 16 weeks:**  -FAST: IPFD ↓ from 12.9% to 11.3% (Δ−1.6 p.p.)  -FED: IPFD ↓ from 12.9% to 12.5% (Δ−0.4 p.p.)  Between-group difference not significant (p=0.521) |
| **Combined diet and exercise interventions** | | | | | | | |
| Vogt et al.^18^ (2016) | Germany | 29 obese adults with T2DM (median age 59.0y; median BMI 34.0 kg/m^2^; 65.5% women) | **- Standardized 15-week weight-loss program:**  - Weeks 0–6: Formula diet (OPTIFAST), ~800 kcal/day; 96 g CHO, 70 g protein, 15 g fat/day  - Weeks 6–10: Structured refeeding phase, caloric intake increased to 1200 kcal/day  - Weeks 10–15: Gradual increase to individually tailored maintenance calories  **- Weekly supervised exercise sessions (combined cardio + strength) + dietitian support** | 15 weeks | PP | MRI (3.0 T) using confounder-adjusted chemical-shift–encoded PDFF; multi-echo sequence (3 echoes); ROIs in pancreas (head, body, tail); mean PDFF (%) computed | Relative to baseline [IPFD: median 9.0% (IQR 7.3 to 18.4)]:  **At 6 weeks (ΔBW -10.0 kg [median]):** IPFD: median 10.2% (IQR 7.2 to 16.5); unchanged Δ+0.7 (median; -1.9 to 2.8; p=0.697)  **At 15 weeks (ΔBW -13.2 kg [median]):** IPFD: median 9.0% (IQR 7.2 to 13.2); unchanged Δ+0.7 (median; -3.4 to 1.6; p=0.820) |
| Zhang et al.^11^ (2024) | China | 53 adults with overweight/obesity and T2DM completed intervention (mean BMI 29.55 kg/m^2^; 39 underwent MRI) | **– 500 cal/day CR diet**  -**Exercise 5×/week + sham acupuncture** | 5 weeks | PP | MRI (3.0T), GE Discovery MR750; IDEAL-IQ/PDFF acquisition; three 150-mm² ROIs in the pancreas, avoiding adjacent structures and main vessels; mean of three ROIs used as pancreatic fat (%) | **At 5 weeks:**  Control/sham arm: IPFD ↓ from 12.14% to 11.52% (Δ −0.62 p.p.) |
| Liu H et al.^22^ (2025) | China | 104 adults with obesity who completed follow-up (median age 40.8; BMI ≥28 kg/m²; 48.1% women) | Structured lifestyle program combining:  **Caloric-restricted balanced diet (energy intake:** TEE − 500 kcal/day; 45–55% CHO, 15–20% protein, 20–30% fat)  **Exercise program:** ≥150 min/week of moderate aerobic exercise + ≥2 resistance sessions/week  Monthly follow-ups + daily remote monitoring (WeChat groups)  No glucose-lowering medications used throughout the study | 26 weeks | PP | MRI (3.0T), Philips Ingenia Elition X; multi-echo Dixon sequence; pancreatic PDFF (%) from three 100 mm² ROIs (head, body, tail), averaged | **At 6 months (ΔBW 81.6 kg to 75.0 kg; medians):** IPFD ↓ from 8.8% (median; IQR 5.0 to 14.4) → 5.8% (median; IQR 4.0 to 8.2) (Δ-3.0 p.p., ~34.1% relative decrease, p<0.001) |
| Cheng et al.^23^ (2026) | China | 23 adults with obesity who completed follow-up (mean age 31.9; mean BMI 34 kg/m²; 56.5% women) | **CRD:** BMR × activity factor −500 kcal/day; 40% CHO, 25% protein, 35% fat  **Exercise:** brisk walking 30–60 min/session, 5–7×/week + resistance exercise 2×/week + lifestyle modifications | 24 weeks | PP | MRI (3.0T), six-point Dixon VIBE PDFF; whole-pancreas manual segmentation on PDFF maps; mean pancreatic fat fraction (%) | **At 24 weeks:**  CRD: IPFD ↓ from 14.1±7.2% to 10.5±6.2% (Δ−3.6 p.p.; ~25.5% relative decrease) |
| **Other lifestyle interventions** | | | | | | | |
| Cadenas-Sanchez et al.^20^ (2022) | Spain | 101 children with overweight/obesity included in per-protocol analysis | **- Exercise program (Aerobic + resistance training combined + psychoeducation):** 3–5 sessions/week; 90 minutes; moderate-to-vigorous (MVPA); games, circuits, interval aerobic training, strength exercises; **supervised** by exercise physiologists  **- Control group (psychoeducation only):** maintained usual daily lifestyle; no structured exercise | 20 weeks | PP, ITT | MRI (3.0T), Siemens Magnetom; multi-echo Dixon (mDIXON) sequence; automated segmentation using validated software; pancreatic fat fraction (%) derived voxelwise; whole-pancreas fat fraction obtained by averaging across all slices | **At 20 weeks:**  - Exercise + psychoeducation group: IPFD unchanged from 3.2% → 2.6% (Δ-0.6 p.p., ~18.8% relative decrease)  - Psychoeducation group: IPFD unchanged from 3.2% → 2.9% (Δ-0.3 p.p., ~9.4% relative decrease) |

**Table S3.** Absolute change in MRI-measured IPFD by intervention type.

| **Type of intervention** | **Studies, n** | **Patients, n** | **MD, %** | **95% confidence interval** | **I², %** | **τ²** | **Subgroup difference** |
| --- | --- | --- | --- | --- | --- | --- | --- |
| Diet alone | 12 | 577 | -1.04 | -1.31 to -0.78 | 0 | 0 | 0.068 |
| Exercise alone | 3 | 88 | -1.29 | -1.60 to -0.97 | 0 | 0 |  |
| Diet and exercise combined | 4 | 195 | -2.46 | -4.74 to -0.19 | 26.1 | 0.90 |  |

**Table S4.** Leave-one-out sensitivity analysis.

|  | **Studies, n** | **Patients, n** | **SMD** | **95% confidence interval** | **I^2^, %** | **τ^2^** |
| --- | --- | --- | --- | --- | --- | --- |
| **Overall** | **23** | **1036** | **-0.26** | **-0.36 to -0.16** | **12.9** | **0.01** |
| Omitting Lim et al. (2011) | 22 | 1025 | -0.26 | -0.36 to -0.16 | 15.0 | 0.02 |
| Omitting Rossi et al. (2012) | 22 | 1012 | -0.25 | -0.35 to -0.15 | 14.5 | 0.02 |
| Omitting Steven et al. (2016) | 22 | 1007 | -0.25 | -0.35 to -0.15 | 15.9 | 0.02 |
| Omitting Langleite et al. (2016) | 22 | 1014 | -0.26 | -0.36 to -0.16 | 17.0 | 0.02 |
| Omitting Vogt et al. (2016) | 22 | 1007 | -0.26 | -0.36 to -0.16 | 15.1 | 0.02 |
| Omitting Taylor et al. (2018) | 22 | 978 | -0.25 | -0.36 to -0.15 | 16.9 | 0.02 |
| Omitting Heiskanen et al. (2018) | 22 | 1003 | -0.26 | -0.36 to -0.15 | 17.0 | 0.02 |
| Omitting Jiang et al. (2019) | 22 | 899 | -0.30 | -0.40 to -0.20 | 2.2 | <0.01 |
| Omitting Skytte et al. (2019) | 22 | 1009 | -0.26 | -0.36 to -0.16 | 14.9 | 0.02 |
| Omitting Dikariyanto et al. (2020) | 22 | 986 | -0.27 | -0.37 to -0.17 | 13.4 | 0.02 |
| Omitting Fortuin-de Smidt et al. (2020) | 22 | 1016 | -0.25 | -0.35 to -0.15 | 14.3 | 0.02 |
| Omitting Thomsen et al. (2022) | 22 | 970 | -0.26 | -0.37 to -0.16 | 16.6 | 0.02 |
| Omitting Li et al. (2022) | 22 | 983 | -0.26 | -0.36 to -0.16 | 15.1 | 0.02 |
| Omitting Pepa et al. (2022) | 22 | 997 | -0.27 | -0.37 to -0.17 | 13.9 | 0.02 |
| Omitting Cadenas-Sanchez et al. (2022) | 22 | 935 | -0.26 | -0.37 to -0.16 | 16.0 | 0.02 |
| Omitting Dokpuang et al. (2023) | 22 | 1012 | -0.25 | -0.35 to -0.15 | 12.9 | 0.01 |
| Omitting Zhang et al. (2024) | 22 | 997 | -0.26 | -0.36 to -0.16 | 14.2 | 0.02 |
| Omitting Sandby et al. (2024) | 22 | 956 | -0.28 | -0.38 to -0.18 | 14.6 | 0.02 |
| Omitting Harvie et al. (2025) | 22 | 1016 | -0.25 | -0.35 to -0.15 | 15.2 | 0.02 |
| Omitting Liu Z et al. (2025) | 22 | 1004 | -0.23 | -0.31 to -0.15 | 0 | <0.01 |
| Omitting Rees et al. (2025) | 22 | 1021 | -0.26 | -0.36 to -0.16 | 17.0 | 0.02 |
| Omitting Liu H et al. (2025) | 22 | 932 | -0.22 | -0.32 to -0.13 | 0 | <0.01 |
| Omitting Cheng et al. (2026) | 22 | 1013 | -0.25 | -0.35 to -0.15 | 13.6 | 0.02 |

**SUPPLEMENTARY REFERENCES**

1. Steven S, Hollingsworth KG, Al-Mrabeh A, et al. Very low-calorie diet and 6 months of weight stability in type 2 diabetes: pathophysiological changes in responders and nonresponders. *Diabetes Care*. 2016;39(5):808-815. doi:10.2337/dc15-1942

2. Taylor R, Al-Mrabeh A, Zhyzhneuskaya S, et al. Remission of human type 2 diabetes requires decrease in liver and pancreas fat content but is dependent upon capacity for β cell recovery. *Cell Metab*. 2018;28(4):547-556.e3. doi:10.1016/j.cmet.2018.07.003

3. Heiskanen MA, Motiani KK, Mari A, et al. Exercise training decreases pancreatic fat content and improves beta cell function regardless of baseline glucose tolerance: a randomised controlled trial. *Diabetologia*. 2018;61(8):1817-1828. doi:10.1007/s00125-018-4627-x

4. Skytte MJ, Samkani A, Petersen AD, et al. A carbohydrate-reduced high-protein diet improves HbA1c and liver fat content in weight stable participants with type 2 diabetes: a randomised controlled trial. *Diabetologia*. 2019;62(11):2066-2078. doi:10.1007/s00125-019-4956-4

5. Dikariyanto V, Smith L, Francis L, et al. Snacking on whole almonds for 6 weeks improves endothelial function and lowers LDL cholesterol but does not affect liver fat and other cardiometabolic risk factors in healthy adults: the ATTIS study, a randomized controlled trial. *Am J Clin Nutr*. 2020;111(6):1178-1189. doi:10.1093/ajcn/nqaa100

6. Fortuin-de Smidt MC, Mendham AE, Hauksson J, et al. Effect of exercise training on insulin sensitivity, hyperinsulinemia and ectopic fat in black South African women: a randomized controlled trial. *Eur J Endocrinol*. 2020;183(1):51-61. doi:10.1530/EJE-19-0957

7. Thomsen MN, Skytte MJ, Samkani A, et al. Dietary carbohydrate restriction augments weight loss-induced improvements in glycaemic control and liver fat in individuals with type 2 diabetes: a randomised controlled trial. *Diabetologia*. 2022;65(3):506-517. doi:10.1007/s00125-021-05628-8

8. Li M, Zheng Q, Miller JD, et al. Aerobic training reduces pancreatic fat content and improves β‐cell function: A randomized controlled trial using IDEAL‐IQ magnetic resonance imaging. *Diabetes Metab Res Rev*. 2022;38(4). doi:10.1002/dmrr.3516

9. Della Pepa G, Brancato V, Costabile G, et al. An isoenergetic multifactorial diet reduces pancreatic fat and increases postprandial insulin response in patients with type 2 diabetes: a randomized controlled trial. *Diabetes Care*. 2022;45(9):1935-1942. doi:10.2337/dc22-0605

10. Dokpuang D, Zhiyong Yang J, Nemati R, et al. Magnetic resonance study of visceral, subcutaneous, liver and pancreas fat changes after 12 weeks intermittent fasting in obese participants with prediabetes. *Diabetes Res Clin Pract*. 2023;202:110775. doi:10.1016/j.diabres.2023.110775

11. Zhang Z, Bao K, Liu J, et al. Acupuncture-assisted lifestyle intervention improve the metabolic status and spontaneous brain activity of type 2 diabetes Mellitus patients: a randomized, clinical trial. *Diabetol Metab Syndr*. 2024;16(1):255. doi:10.1186/s13098-024-01489-4

12. Sandby K, Magkos F, Chabanova E, et al. The effect of dairy products on liver fat and metabolic risk markers in males with abdominal obesity – a four-arm randomized controlled trial. *Clinical Nutrition*. 2024;43(2):534-542. doi:10.1016/j.clnu.2023.12.018

13. Harvie M, Coe P, Higham C, et al. A randomised controlled trial of short-term intermittent energy restriction [ier] versus continuous energy restriction [cer] on body fat stores and measures of insulin resistance in women with obesity at increased risk of breast cancer. *BMC Nutr*. 2025;11(1):199. doi:10.1186/s40795-025-01181-4

14. Rees JL, Walesiak D, Thompson R, Mager D, Senior P, Boulé NG. HbA1c and liver fat after 16 weeks of fasted versus fed exercise training in adults with type 2 diabetes. *Med Sci Sports Exerc*. 2025;57(1):106-114. doi:10.1249/MSS.0000000000003552

15. Lim EL, Hollingsworth KG, Aribisala BS, Chen MJ, Mathers JC, Taylor R. Reversal of type 2 diabetes: normalisation of beta cell function in association with decreased pancreas and liver triacylglycerol. *Diabetologia*. 2011;54(10):2506-2514. doi:10.1007/s00125-011-2204-7

16. Rossi AP, Fantin F, Zamboni GA, et al. Effect of moderate weight loss on hepatic, pancreatic and visceral lipids in obese subjects. *Nutr Diabetes*. 2012;2(3):e32-e32. doi:10.1038/nutd.2012.5

17. Langleite TM, Jensen J, Norheim F, et al. Insulin sensitivity, body composition and adipose depots following 12 w combined endurance and strength training in dysglycemic and normoglycemic sedentary men. *Arch Physiol Biochem*. 2016;122(4):167-179. doi:10.1080/13813455.2016.1202985

18. Vogt LJ, Steveling A, Meffert PJ, et al. Magnetic resonance imaging of changes in abdominal compartments in obese diabetics during a low-calorie weight-loss program. *PLoS One*. 2016;11(4):e0153595. doi:10.1371/journal.pone.0153595

19. Jiang Y, Spurny M, Schübel R, et al. Changes in pancreatic fat content following diet-induced weight loss. *Nutrients*. 2019;11(4):912. doi:10.3390/nu11040912

20. Cadenas-Sanchez C, Cabeza R, Idoate F, et al. Effects of a family-based lifestyle intervention plus supervised exercise training on abdominal fat depots in children with overweight or obesity. *JAMA Netw Open*. 2022;5(11):e2243864. doi:10.1001/jamanetworkopen.2022.43864

21. Liu Z, Feng N, Wang S, et al. Low-calorie diets and remission of type 2 diabetes in Chinese: phenotypic changes and individual variability. *Nutr J*. 2025;24(1):42. doi:10.1186/s12937-025-01101-z

22. Liu H, Duan J, Guan G, Rong P, Jin P. Effect of lifestyle intervention on the mobilization of fat depots and organ iron deposition in individuals with obesity: a prospective study. *Diabetes, Metabolic Syndrome and Obesity*. 2025;Volume 18:4113-4125. doi:10.2147/DMSO.S564591

23. Cheng H, Jiang X, Zhu X, et al. Effect of calorie restricted diet versus liraglutide on intrapancreatic fat deposition in people with obesity: a pilot study. *Obesity*. 2026;34(4):829-838. doi:10.1002/oby.70153
